# Airborne fine particulate matter exposure induces transcriptomic alterations resembling asthmatic signatures: insights from integrated omics analysis

**DOI:** 10.1093/eep/dvae026

**Published:** 2025-01-02

**Authors:** Daniel González, Alexis Infante, Liliana López, Danilo Ceschin, María José Fernández-Sanchez, Alejandra Cañas, Carlos Zafra-Mejía, Adriana Rojas

**Affiliations:** Institute of Human Genetics, School of Medicine, Pontificia Universidad Javeriana, Bogotá 110231, Colombia; School of Engineering, Universidad Nacional de Colombia, Bogotá 111321, Colombia; Department of Statistics, Universidad Nacional de Colombia, Bogotá 111321, Colombia; Instituto Universitario de Ciencias Biomédicas de Córdoba (IUCBC), Córdoba X5016KEJ, Argentina; Centro de Investigación en Medicina Traslacional “Severo R. Amuchástegui” (CIMETSA), Consejo Nacional de Investigaciones Científicas y Técnicas (CONICET), Córdoba X5016KEJ, Argentina; School of Medicine, Pontificia Universidad Javeriana, Bogotá 110231, Colombia; Pulmonary Unit, Hospital Universitario San Ignacio, Bogotá 110231, Colombia; School of Medicine, Pontificia Universidad Javeriana, Bogotá 110231, Colombia; Pulmonary Unit, Hospital Universitario San Ignacio, Bogotá 110231, Colombia; Grupo de Investigación en Ingeniería Ambiental (GIIAUD), Facultad del Medio Ambiente y Recursos Naturales, Universidad Distrital Francisco José de Caldas, Bogotá 110321, Colombia; Institute of Human Genetics, School of Medicine, Pontificia Universidad Javeriana, Bogotá 110231, Colombia; Department of Genetics, University of Córdoba, Córdoba 14071, Spain; Maimónides Biomedical Research Institute of Córdoba (IMIBIC), Córdoba 14004, Spain; Reina Sofía University Hospital, Córdoba 14004, Spain

**Keywords:** environment, epigenetics, transcriptome, air pollution, asthma, risk factor

## Abstract

Fine particulate matter (PM_2.5_), an atmospheric pollutant that settles deep in the respiratory tract, is highly harmful to human health. Despite its well-known impact on lung function and its ability to exacerbate asthma, the molecular basis of this effect is not fully understood. This integrated transcriptomic and epigenomic data analysis from publicly available datasets aimed to determine the impact of PM_2.5_ exposure and its association with asthma in human airway epithelial cells. Differential gene expression and binding analyses identified 349 common differentially expressed genes and genes associated with differentially enriched H3K27ac regions in both conditions. Co-expression network analysis revealed three preserved modules (Protein Folding, Cell Migration, and Hypoxia Response) significantly correlated with PM_2.5_ exposure and preserved in asthma networks. Pathways dysregulated in both conditions included epithelial function, hypoxia response, interleukin-17 and TNF signaling, and immune/inflammatory processes. Hub genes like TGFB2, EFNA5, and PFKFB3 were implicated in airway remodeling, cell migration, and hypoxia-induced glycolysis. These findings elucidate common altered expression patterns and processes between PM_2.5_ exposure and asthma, helping to understand their molecular connection. This provides guidance for future research to utilize them as potential biomarkers or therapeutic targets and generates evidence supporting the need for implementing effective air quality management strategies.

## Introduction

Air pollution, a significant environmental health concern, is linked to a spectrum of chronic respiratory, cardiovascular, metabolic, and other debilitating diseases. Among air pollutants, fine particulate matter (PM_2.5_) poses a significant threat to human health. PM_2.5_, a complex mixture of solid and liquid particles, can penetrate deep into the lungs, inducing inflammation, oxidative stress, and other detrimental effects [1, 2]. Despite well-documented adverse health impacts, the molecular mechanisms underlying the detrimental effects of PM_2.5_ exposure remain elusive.

A 2019 study suggested that up to 4 million new pediatric asthma cases (13% global incidence) may be attributable to air pollution exposure [3]. Asthma, a prevalent and heterogeneous disease, is characterized by chronic airway inflammation. Its prevalence among adults in 17 countries averages 4.4%, ranging from 0.9% to 29.0% [4]. Asthma is defined by the history of respiratory symptoms, such as wheeze, shortness of breath, chest tightness, and cough, which vary over time and in intensity, together with variable expiratory airflow limitation [5]. T2 asthma, an endotype/phenotype of asthma, is characterized by a T2-mediated inflammatory response. T2 cells produce pro-inflammatory cytokines, such as interleukin-4 (IL-4), interleukin-5 (IL-5), and interleukin-13 (IL-13), which promote mucus production, inflammatory cell recruitment, and airway remodeling [5, 6].

Recent studies have shown that air pollution directly affects epigenetic mechanisms, influencing gene expression associated with respiratory, cardiac, and tumor pathologies. For example, the Liu group reported changes in H3K27ac levels in individuals exposed to PM_2.5_ [7]. It is well established that different histone marks have distinct roles and patterns in the genome. H3K27ac, in particular, is specifically enriched in active enhancers and promoters, making it a valuable marker for identifying active genomic regions in epigenetic studies [8]. Characterizing the effects of environmental conditions on gene expression using high-throughput sequencing technologies, such as messenger RNA sequencing (RNA-seq) or chromatin immunoprecipitation sequencing (ChIP-seq), can aid in elucidating the molecular mechanisms disrupted by exposure to unfavorable environmental conditions. This approach can foster a deeper understanding of complex disease development and facilitate the identification of gene meta-signatures associated with these diseases or potential biomarkers.

The development of high-throughput sequencing technologies, like RNA-seq or ChIP-seq, has brought a large availability of omics data. Those are frequently used in research and stored on databases like The Gene Expression Omnibus, from the NCBI [9]. Through re-analyses approaches it is possible to find information that was not contemplated on the research questions formulated by the original authors. Successful re-analysis has been done before to identify biomarkers and other relevant aspects related to asthma [10–12]. New knowledge generated through these analyses can also be used to propose biological hypotheses, gain a deeper understanding of biological processes and therefore make better public health decisions.

Traditional analysis methods over RNA-seq and ChIP-seq data analysis are highly focused on individual genes. Essentially, differential gene expression analysis helps to find changes in expression levels between experimental groups, like disease versus control groups, in a quantitative way [13]. Similarly, through differential enriched region analysis in ChIP-seq assays and subsequently peak annotation, genes influenced by proteins that interact with DNA, like posttranslational modified histones, could be identified [14]. Thus, interactions between genes go unnoticed through traditional workflows [15]. RNA-seq and ChIP-seq data integration is essential for elucidating transcriptional regulation mechanisms. This approach reveals not only the genes affected by specific exposures or diseases but also the potential epigenetic machinery mediating these effects. Other authors have successfully integrated these data sources to gain a better understanding of the transcriptional landscape in other organisms [16, 17].

Gene co-expression networks help to elucidate gene interactions and thus propose gene meta-signatures or biomarkers from the systemic level and thus complement other analyses. This approximation is supported by the idea that proteins play their functions through interactions with others and is built from the similarities in expression levels between genes. Phenotype-specific gene co-expression network comparison is based on the conception of differential networking and helps to uncover specific rewiring patterns induced by phenotypes, which could be assessed through node topological properties and centrality measures [15, 18]. The genes of this network should be key genes identified by previous analysis. In this study, we prioritized the genes to be included in the network-integrating transcriptomic data and ChIP-seq data.

To investigate the effects of PM_2.5_ exposure on the human airway epithelial cells (AECs) transcriptome and its potential relevance to asthma, we conducted an omics integration of four RNA-seq datasets and two ChIP-seq datasets available at the Gene Expression Omnibus and Sequence Read Archive Databases. Our aim was to identify the shared transcriptomic and H3K27ac epigenomic signature profiles between PM_2.5_ exposure and asthma diagnosis conditions.

## Results

### Identified DEGs/GADERs common to PM_2.5_-exposed healthy subjects and asthmatics

To identify potential similarities in the transcriptomic and epigenomic profiles induced by PM_2.5_ exposure and those observed in asthma, an integrative *in silico* analysis of publicly available RNA-seq and ChIP-seq datasets was conducted. Genes exhibiting inconsistent regulatory patterns and expression profiles across datasets were excluded from further analyses.

For PM_2.5_ exposure, four datasets comparing exposed and nonexposed cells were selected, yielding a total of 3183 identified genes, 1570 upregulated and 1613 downregulated genes (Fig. 2a and b). In the four asthma diagnosis datasets contrasting nonasthmatic and asthmatic cells, a total of 4044 genes were detected: 2067 upregulated and 1977 downregulated genes (Fig. 2c and d). The genes located at the intersection of the Venn diagrams (90 and 52) correspond to genes with discordant expression patterns that were excluded from the analysis (Fig. 2b and d).

**Figure 1.**
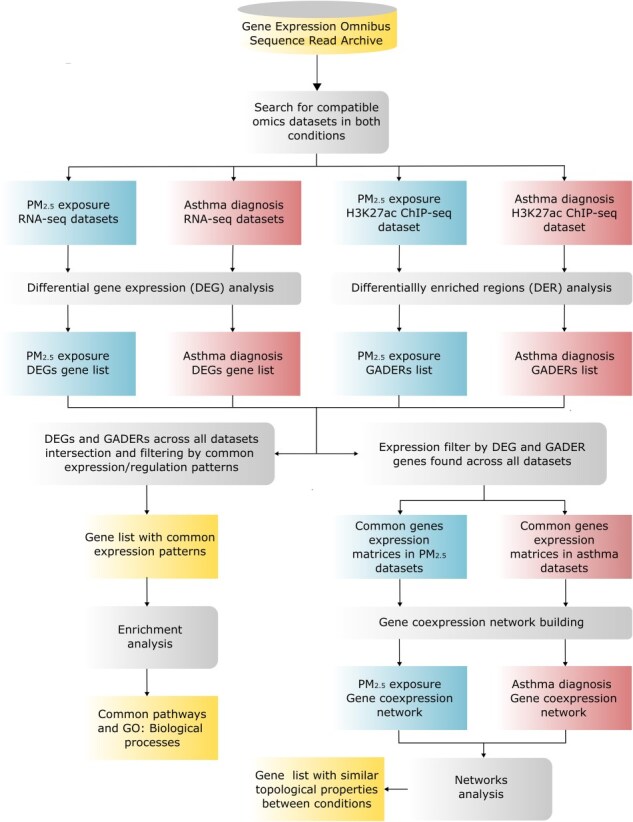
Bioinformatics data analysis pipeline. Schematic representation of the step-by-step workflow for analyzing RNA-seq and ChIP-seq data, starting from the raw files obtained from the NCBI and culminating in the detection of DEGs and differentially enriched regions (GADERs) for downstream analysis, and networks analysis.

**Figure 2.**
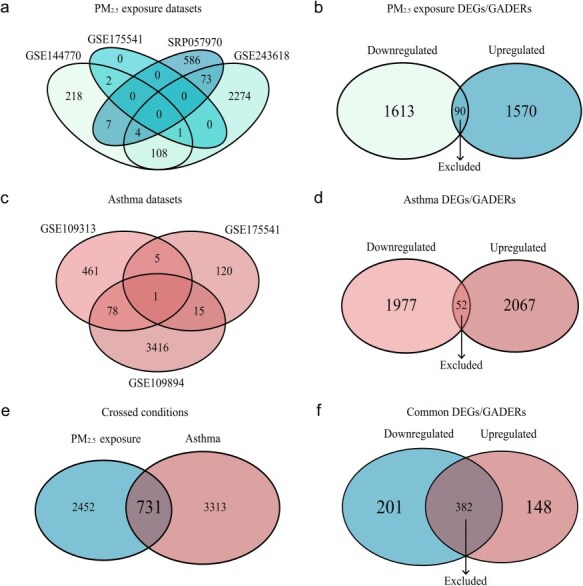
Common epigenetic and transcriptomic signatures induced by air pollution exposure and asthma. This study investigates the impact of air pollution exposure on epigenetic and transcriptomic changes, specifically concerning asthma. Data from four datasets related to PM_2.5_ exposure (a) were utilized, resulting in the identification of 3183 DEGs/GADERs after excluding discordant ones (intersection) (b). Additionally, asthma-related data from three datasets (c) were analyzed, leading to the discovery of 4044 DEGs/GADERs after excluding discordant ones (intersection) (d). The intersection of DEGs/GADERs from both conditions revealed 731 common DEGs/GADERs (e), among which 349 exhibited shared expression/regulation patterns (f).

The intersection of total DEGs/GADERs from both conditions revealed 731 common DEGs/GADERs, among which 349 exhibited similar expression/regulation patterns; therefore, being chosen to focus on in subsequent analyses (Fig. 2e and f; Table 1). A comprehensive list of the 148 upregulated and 201 downregulated DEGs/GADERs shared between PM_2.5_ exposure and asthma diagnosis, along with their respective magnitudes of expression fold change against controls is provided in Supplementary Table S1.

**Table 1.** Characterization of selected public datasets for omics integration

Geo accession	Condition	Cells	Subjects, case/control	Analysis	Number of detected degs/gaders
GSE144770	High PM_2.5_ exposure	HNECs	4/4	RNA-seq PE	340 DEGs
GSE175541	High PM_2.5_ exposure	HNECs	4/4	RNA-seq PE	3 DEGs
GSE243618	High PM_2.5_ exposure	HNECs	3/3	RNA-seq PE	2460 DEGs
Total DEGs associated to PM_2.5_ exposure					2644 DEGs
SRP057970	High PM_2.5_ exposure	PMNs	2/2	ChIP-seq SE	670 GADERs
Total DEGs/GADERs associated to PM_2.5_ exposure					3183 DEGs/GADERs
GSE109313	Atopic asthma diagnosis	HNECs	9/10	RNA-seq PE	547 DEGs
GSE144770	Asthma GINA diagnosed	HNECs	4/4	RNA-seq SE	0 DEGs
GSE175541	Asthma GINA diagnosed	HNECs	4/4	RNA-seq PE	141 DEGs
Total DEGs associated to asthma diagnosis					678 DEGs
GSE109894	Asthma	HBECs	4/3	ChIP-seq SE	3510 GADERs
Total DEGs/GADERs associated to asthma diagnosis					4044 DEGs/GADERs
Total common DEGs/GADERs between PM_2.5_ exposure and asthma diagnosis					349 DEGs/GADERs

Dataset identifiers, sample size, experimental conditions, data types (e.g. transcriptomic and epigenetic), and relevant findings, these datasets were curated and integrated to provide a comprehensive analysis of the molecular signatures associated with the studied conditions; therefore, all totals showed eliminated genes with discordant expression patterns across datasets. Abbreviations: HBECs: human bronchial epithelial cells; HNECs: human nasal epithelial cells; PMNs: polymorphonuclear leukocytes; PE: paired-end; SE: single-end.

### Functional annotation analysis of identified common DEGs/GADERs

**Figure 3.**
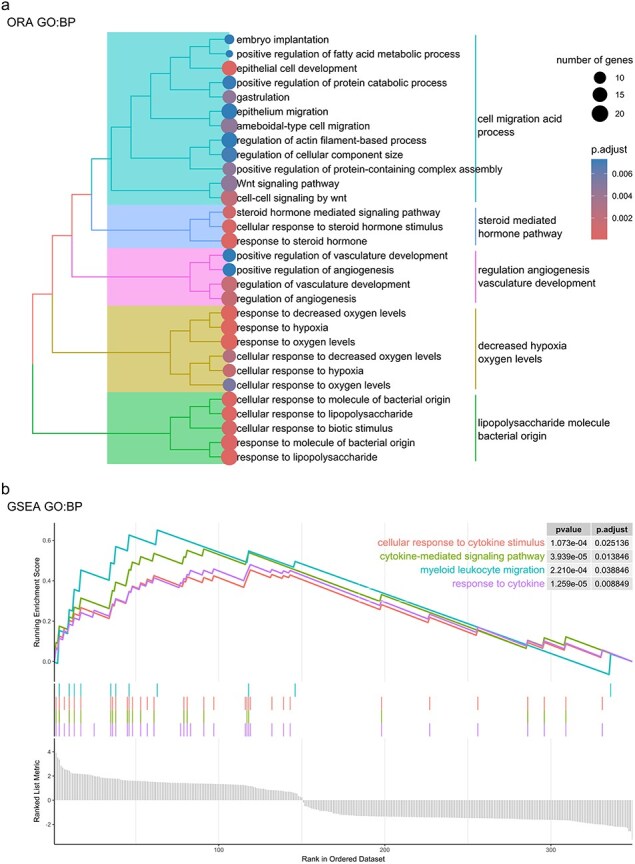
Biological processes associated with common DEGs/GADERs found between PM_2.5_ exposure and asthma. (a) Treeplot displaying hierarchical clustering based on pairwise similarity among the 152 biological processes identified via ORA. Each subtree is labeled with high-frequency words summarizing the cluster’s characteristics. (b) GSEAplot illustrating the differential enrichment of biological processes shared between PM_2.5_ exposure and asthma. The *y*-axis quantifies the ranking metric, while the *x*-axis ranks all genes by the mean log2FC from both conditions. The lower section of the plot shows the ranked gene list. The upper section highlights the gene set’s enrichment score (ES), which peaks at the plot’s apex.

To elucidate the biological processes linked with the identified common DEGs/GADERs, an over-representation analysis (ORA) was performed, yielding 152 associated biological processes (Supplementary Table S2). To reduce the complexity and ease the interpretation of these enrichment results, a hierarchical clustering based on term similarity was conducted, resulting in the identification of five clusters encompassing multiple terms related to epithelium processes, steroid hormone signaling, angiogenesis, hypoxia, and (lipopolysaccharide) LPS response (Fig. 3a, Supplementary Fig. S1a). The specific genes and their expression patterns from the top 25 enriched terms associated with epithelium processes, hypoxia, and immune response are presented in Supplementary Fig. S2.

In addition, ORA for KEGG pathways revealed a statistically significant overrepresentation of these genes belonging to four pathways: TNF signaling pathway (*P*_adj_ = .003), IL-17 signaling pathway (*P*_adj_ = .011), rheumatoid arthritis (*P*_adj_ = .039), and PPAR signaling pathway (*P*_adj_ = .041). These pathways included 22 related genes, notably, genes such as *CSF1, CXCL2, CXCL3, CXCL8, FOS, IL1B, PTGS2*, and *TNFAIP3* were all upregulated and present in more than one enriched pathway (Supplementary Fig. S1b).

Interestingly, gene set enrichment analysis (GSEA) for the common DEGs/GADERs identified a statistically significant association of the fold change of these genes with four terms: cellular response to cytokine stimulus (*P*_adj_ = .025), cytokine-mediated signaling pathway (*P*_adj_ = .014), myeloid leukocyte migration (*P*_adj_ = .039), and response to cytokine (*P*_adj_ = .009) (Fig. 3b). These biological processes had 27 related genes, and remarkably, the genes *CSF1, CSF3, CXCL2, CXCL3, CXCL8, CYP1B1, IL1B, IL1A, EREG, IL1RN, TNFAIP3, IL2RG, GBP1, CTR9, NFKBIZ, IFNGR1, UGCG, VAMP3, KLF4, EGR1, EDN1*, and *FOS* were all upregulated and found in more than one biological process (Supplementary Fig. S1C).

A comprehensive ORA analysis was conducted using the g:OST tool to identify potential regulators of the common DEGs/GADERs. The analysis revealed that 55 genes (15.8%) are targeted by the miRNA hsa-miR-124-3p (MIR124-1) (*P*_adj_ = 8.86 × 10^−03^). Furthermore, regulatory motifs were identified for transcription factors *SP1, TFAP2A, TRIM28, ETF, EGR2, CTCF, ZBTB33, WT1*, and *CHURC1* on 241, 138, 132, 183, 113, 131, 218, 148, and 238 of the common DEGs/GADERs, respectively. These associations all demonstrated adjusted p-values lower than 1 × 10^−07^.

### Co-expression network analysis

To evaluate expression patterns from a systemic perspective and uncover relationships between genes across evaluated conditions, expression profiles of genes identified through differential gene expression or differential enriched region analyses were used. These expression profiles served as input for co-expression networks construction, one per evaluated condition. After filtering low expressed genes, a total of 2724 genes were used for the construction of the co-expression networks. Soft thresholds for PM_2.5_ and asthma networks were set at powers of 17 and 12, respectively (Supplementary Fig. S3).

**Figure 4.**
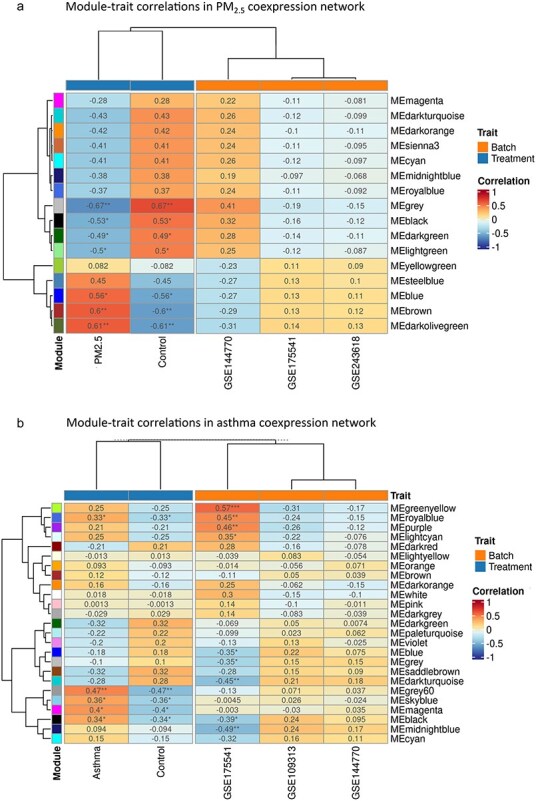
Module-trait correlation heatmaps. Module-trait correlation heatmaps in (a) PM_2.5_ exposure and (b) asthma co-expression networks effect in the orange columns are represented. Significance levels are indicated by asterisks. *P-value < .05; **P-value = .01; ***P-value < .001. All modules identified in the asthma network are directly correlated with the asthma condition, while in PM_2.5_ networks three modules are directly correlated with the exposure and four inversely correlated.

**Figure 5.**
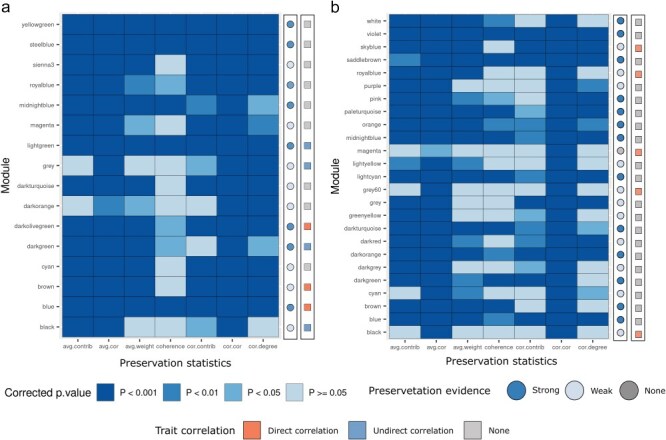
Module preservation analysis. Module preservation analysis heatmaps in (a) PM_2.5_ exposure and (b) asthma co-expression networks. Each row represents a co-expression module, and each column represents a preservation statistic: average node contribution (avg.contrib), density of correlation structure (avg.cor), average edge weight (avg.weight), module coherence (coherence), concordance of node distribution (cor.contrib), concordance of correlation structure (cor.cor), concordance of weight degree (cor.degree). Tiles are colored according to the corrected *P*-value. Two additional columns are presented on the right side: the first summarizes module preservation and the second indicates if the module is correlated with its respective trait.

**Figure 6.**
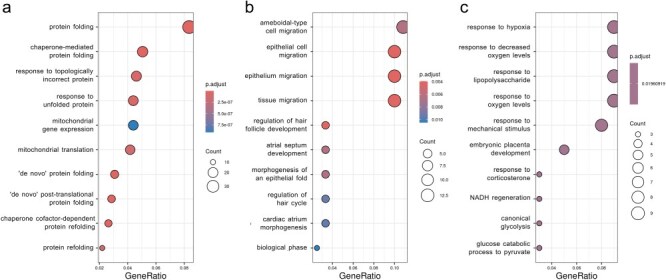
Preserved modules enrichment analyses. Enrichment analyses performed with each set of genes in modules preserved across co-expression networks. The enrichment analyses were performed over biological process terms in the Gene Ontology database. The figure part (a) corresponds to the first in the text. The figure part (b) corresponds to the second in the text. The figure part (c) corresponds to the third in the text. Each row represents a term, the size of the dot represents the number of genes associated with the respective term and the position of the dot on the horizontal axis represents its significance.

Hierarchical clustering and dynamic tree cut identified 25 modules of highly co-expressed genes in the asthma co-expression network and 18 modules in the PM_2.5_ network (Supplementary Fig. S4). As shown in Fig. 4, in the asthma diagnosis co-expression network five modules were significantly correlated with the asthma diagnosis condition. On the other hand, in the PM_2.5_ network, three modules were significantly associated with the PM_2.5_ exposure and four with the control condition. Module preservation analyses across networks showed three modules significantly correlated with PM_2.5_ exposure significantly preserved in the asthma diagnosis network (Fig. 5). Enrichment analysis over member genes for each preserved module allowed the identification of three labels: Protein Folding, Cell Migration, and Hypoxia Response (Fig. 6). Total hub genes in preserved modules are indicated in Supplementary Table S3.

**Figure 7.**
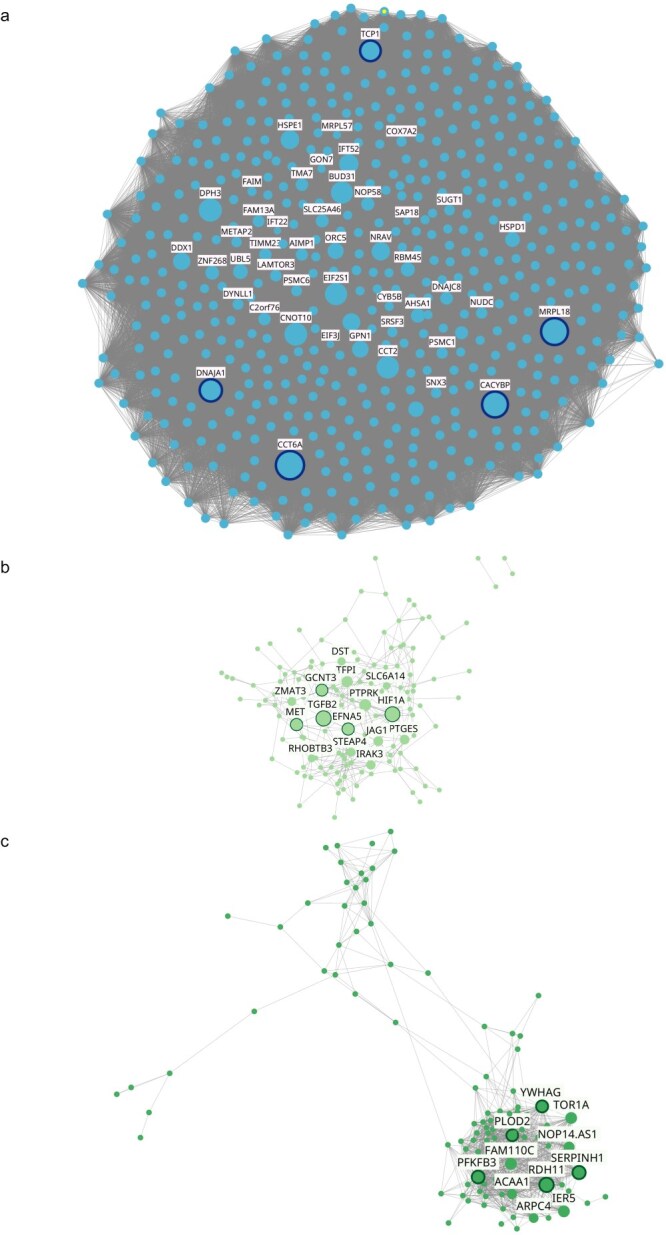
Preserved co-expression modules. Co-expression modules identified in the PM_2.5_ exposure co-expression network significantly correlated with the exposure to PM_2.5_ and preserved into the asthma network. Illustrated modules were annotated as being related to protein folding (a), cell migration (b), and hypoxia response (c). Each node represents a gene and each edge a co-expression relationship. The names of all identified hub genes are shown, with the top five hub genes, based on intramodular connectivity, highlighted with a darker outer circle. Additionally, the size of each node reflects its intramodular connectivity.

The Protein Folding module, shown in Fig. 7a, had 510 genes and 109 860 edges. Inside this module, the top five hub genes with the highest connectivity were related to chaperon complexes: CCT6 [19], MRPL18 [20], DNAJA1 [21, 22], TCP1 [19], and CACYBP [23]. Additionally, MRPL18 [20] and DNAJA1 [22] were reported to be related to mitochondrial transport and CACYB with calcium-dependent ubiquitination and subsequently proteasomal degradation of target proteins, where beta-catenin (CTNNB1) is reported among those. DNAJA1 [24] and CACYBP [25] were also reported to participate in stress response.

The second module, annotated as Cell Migration, is illustrated in Fig. 7b. In this module, 140 genes and 395 edges were identified. Most of the top hub genes were identified as key elements in regulation processes. TGFB2 and MET encode growth factor proteins and participate in epithelial remodeling, cell migration, cell proliferation, morphogenesis, and survival [26–29]. HIF1A encodes the alpha subunit of transcription factor hypoxia-inducible factor 1 (HIF-1), a master regulator of cellular and systemic response to hypoxia [30]. EFNA5 is a cell surface GPI-bound ligand for Eph receptors, a family of tyrosine kinase receptors that are crucial for migration, repulsion, and adhesion during epithelial development [31]. GCNT3, also a top hub gene, encodes a glycosyltransferase that participates in mucin biosynthesis; mucins are glycoproteins involved in cell–cell recognition, immune response, carcinogenesis, and tumor metastasis [32].

The third module founded in preservation analysis, annotated as Hypoxia Response, had 110 genes and 1072 edges. This module is presented in Fig. 7c. Top hub genes had diverse functions. For instance, RDH11 encodes a retinal reductase enzyme that catalyzes the reduction of retinal to retinol; also, PFKFB3 gene belongs to a family of bifunctional proteins that are involved in both the synthesis and degradation of fructose-2,6-bisphosphate, a regulatory molecule that controls glycolysis in eukaryotes [33]; PLOD2 and SERPINH1 are involved in collagen synthesis, PLOD2 encodes a membrane-bound homodimeric enzyme that catalyzes the hydroxylation of lysyl residues in collagen/like peptides, plays an important role in the stability of collagen cross-links and the formation of normal mature collagen [34]; similarly, SERPINH1 belongs to a family of serine proteinase inhibitors, plays a role in collagen biosynthesis as a collagen-specific molecular chaperone [35]. Finally, YWHAG was reported as the gene with the most diverse functions in this module. YWHAG belongs to an adapter protein family with the ability to interact with serine/threonine phosphorylated target proteins, such as kinases and transcription factors, and thus regulate them by modifying their activity, stability, and localization [36].

## Discussion

This integrative analysis provided novel insights into the common molecular processes deregulated during exposure to PM_2.5_ and the development of asthma. Differential expression analysis and differential binding analysis identified 349 common DEGs and genes under common differentially enriched H3K27ac regions (GADERs) in both PM_2.5_ exposure and T2-high asthma. Co-expression network analysis of identified genes across all datasets revealed three modules significantly correlated with PM_2.5_ exposure and preserved in the asthma network. Asthma is a chronic airway disease marked by inflammation, airway hyperresponsiveness (AHR), and structural changes collectively termed airway remodeling. Herein, biological processes related to epithelial function, hypoxia, and immune/inflammatory responses and pathways such as IL-17 and TNF, which are known to be implicated in asthma pathogenesis, were found to be commonly dysregulated in response to environmental pollutants like PM_2.5_, indicating that these pathways may partially mediate the effects of such exposures on both inflammatory and structural processes in the lungs.

The dysregulation of processes associated with epithelial function is a hallmark of airway remodeling, which encompasses a broad range of changes, including epithelial-to-mesenchymal transition (EMT), subepithelial fibrosis, smooth muscle hypertrophy, and mucus hypersecretion. These changes are driven in part by inflammation-induced tissue injury and subsequent repair processes, leading to airway wall thickening and narrowing, thus contributing to AHR and persistent airflow limitation [37]. Some studies support our findings, addressing that PM_2.5_ exposure might facilitate airway remodeling by inducing the TGF-β/Smad3 pathways, which activate the EMT signaling cascades [38–40]. Regarding the dysregulation of the hypoxia response observed in both PM_2.5_ exposure and asthma, it has been previously reported that PM_2.5_ induces cardiac hypoxia and induces a hypoxia-induced inflammation status [41, 42].

One of the key altered pathways involved in asthma, particularly in severe forms, is the IL-17 pathway. It is well known that IL-17 and IL-17 F can induce lung structural cells to secrete pro-inflammatory cytokines (e.g. TNF, IL-1β, G-CSF, and IL-6) and chemokines (e.g. CXCL1/Gro-α, CXCL2, and CXCL8/IL-8), thereby triggering neutrophil infiltration [43–45]. Herein we show that both PM_2.5_ exposure and asthma exhibited overexpression of proinflammatory cytokines such as IL-1β*, TNFAIP3*, G-CSF, and M-CSF (encoded by *CSF3* and *CSF1* genes), and several members of the CXCL family of chemokines (as *CXCL2, CXCL3, CXCL8*), while showing low expression of *TRAF5*, a gene whose deficiency enhances Th17 cells development [46].

Beyond that well-characterized role in driving neutrophilic inflammation, IL-17 contributes significantly to airway remodeling, by stimulating the production of TGF-β which leads to EMT induction, inducing the release of matrix metalloproteinase 9 (MMP-9), which degrade the extracellular matrix proteins, including collagen and laminin, and promoting the survival of airway smooth muscle (ASM) cells, enhancing their proliferation and contributing to the narrowing of the airway walls. Additionally, IL-17A and IL-17 F stimulate airway epithelial and smooth muscle cells to produce mucins (MUC5B and MUC5AC), enhancing mucus hypersecretion and contributing to airway obstruction [47–49].

Similarly, the TNF signaling pathway has been implicated in both the inflammatory and structural aspects of asthma. TNF-α, a proinflammatory cytokine produced by various immune cells, directly affects ASM by enhancing contractility and promoting AHR. It also stimulates the proliferation of fibroblasts and their differentiation into myofibroblasts, key players in the deposition of extracellular matrix proteins such as collagen, leading to subepithelial fibrosis, a critical component of airway remodeling. Additionally, TNF-α promotes epithelial cell apoptosis and increases the production of adhesion molecules, further facilitating the infiltration of immune cells into the airways [50]. Thus, the disruption of IL-17 and TNF pathways not only drives chronic inflammation but also promotes the structural changes that underpin airway remodeling in asthma.

Several studies support the finding of increased IL-17 signaling in PM_2.5_ exposure [51–53], specifically involving enhanced Th17 cell differentiation and increased IL-17 secretion through activation of the aryl hydrocarbon receptor by polycyclic aromatic hydrocarbons (PAHs) present in PM_2.5_, leading to an inflammatory response and potential exacerbation of lung diseases [53]. On the other hand, increased levels of IL-17 in the airways of asthma patients have been reported, and aberrant IL-17 production is considered a key determinant of severe forms of asthma. This increase might be attributed to the activation of IL-17-producing cells, including Th17 cells, invariant natural killer T cells, mucosal-associated invariant T cells, eosinophils, and macrophages, which secrete chemokines involved in the pathway [49, 54–58].

Th17 cells play a pivotal role in the pathogenesis of asthma, particularly in forms characterized by neutrophilic inflammation, such as steroid-resistant and severe asthma. These cells are a distinct subset of CD4^+^ T helper cells, which primarily secrete IL-17A and IL-17 F. Their main function is to recruit and activate neutrophils, contributing to persistent airway inflammation [59]. Their involvement in asthma is particularly significant in noneosinophilic phenotypes, where Th2-driven inflammation is less prominent; nevertheless, most patients from the integrated studies exhibited a T2-high asthma-like endotype. This might be explained by the recent finding of a hybrid Th17/Th2 cell subset that co-expresses both IL-17 and Th2 cytokines, primarily found in inflamed lungs and elevated in the blood of patients with atopic asthma [60]. These IL-17-producing Th2 cells express both GATA3 and RORγt transcription factors, required for the development of the Th2 and Th17 cell lineages, respectively, remarking the key role of IL-17 in both T2-high and low endotypes [45]. These results suggest that a major mechanism behind asthma development by PM_2.5_ exposure might be the activation of IL-17-producing cells, resulting in the activation of pathways that facilitate a proinflammatory response through leukocyte activation, migration, and survival promotion [45, 61].

Differential gene expression analysis and differential binding analysis often identify key genes affected by conditions like asthma or PM_2.5_ exposure. However, these analyses do not reveal how these genes influence each other or uncover underlying expression patterns. Since genomic elements exert their functions through interactions, employing methods that elucidate these interactions is crucial. Gene co-expression network analysis allows the identification of expression patterns from gene expression data and reveals influence relationships between genes. Consequently, common expression patterns between conditions like asthma and PM_2.5_ exposure are not only limited to which genes are differentially expressed but also include the extent of their expression and their interactions.

The network construction utilized expression profiles of differentially expressed genes across all datasets and genes related to differentially enriched H3K27ac regions from both ChIP-seq datasets. Three modules from the PM_2.5_ co-expression network were significantly preserved in the asthma co-expression network, indicating that their gene expression patterns were not only correlated with PM_2.5_ exposure but also shared with the asthma condition. Module preservation is taken as an indication of biological relevance [62].

Three modules significantly correlated with PM_2.5_ exposure were preserved in the asthma co-expression network. Subsequent module annotation by enrichment analysis of hub genes in each module led to the assignment of three categories: Protein Folding, Cell Migration, and Hypoxia Response. Interestingly, these findings supported previous terms found in enrichment analyses of DEGs and GADERs with similar expression patterns across conditions.

Top hub genes found in the first module, annotated as Protein Folding, were related to chaperone complexes, which are fundamental in protein homeostasis by maintaining a balance between protein synthesis, folding, assembly, and degradation [63]. The CCT6 and TCP1 genes, encoding components of the chaperonin-containing TCP1 complex (TRiC), were identified as top hub genes [19]. TRiC assists in the folding and hydrolysis of ∼10% of cytosolic proteins, including key structural and regulatory proteins like tubulin and actin, which depend on TRiC for their correct folding. Consequently, TRiC is also related to cell proliferation and cell migration processes [64, 65].

The second preserved module was annotated as Cell Migration associated; a biological process indirectly influenced by TRiC activity. Top hub genes identified in this module were directly implicated in gene expression regulation, acting as growth factors or transcription factors. Among these, TGFB2 has been extensively studied in airway epithelial remodeling, cell migration [26–28], and other biological processes like epithelial–mesenchymal transition in cancer [66]. TGFB2 is expressed by eosinophils and promotes profibrotic responses affecting airway remodeling and regulating mucin production [67]. Iwanaga *et al*. reported TGFB2 overexpression in AECs+ cell cultures from asthmatic children but not in control cell cultures exposed to PM_2.5_ [68]. Similarly, TGFB2 was reported as underexpressed in BEAS-2B cells, a human bronchial epithelial cell model, after exposure to different PM_2.5_ mixtures [69].

EFNA5 is another hub gene from the second module, a cell surface GPI-bound ligand for Eph receptors, a family of tyrosine kinase receptors crucial for migration, repulsion, and adhesion during epithelial development. EFNA5 binds promiscuously to Eph receptors on adjacent cells, leading to contact-dependent bidirectional signaling. It has been reported to activate the EPHA3 receptor to regulate cell–cell adhesion, and cytoskeletal organization [31], and is upregulated in astrocytes under stressful conditions [70].

In addition to the Protein Folding module, hub genes DNAJA1 and MRPL18, besides their relation to chaperone complexes, are related to the mitochondrial response to cell stress [71, 72]. Mitochondrial dysfunction has been reported as an important potential injury caused by PM_2.5_ in AECs, leading to increased mitochondrial ROS generation, intracellular calcium levels, and suppression of mitochondrial respiratory function by reducing basal and maximal respiration and ATP production [73]. Mitochondrial dysfunction has also been associated with allergic asthma in terms of calcium homeostasis, oxidative stress, and apoptosis [74].

Hypoxia Response was the main term found in the enrichment analysis of hub genes from the third module and in the ORA analysis of common DEGs/GADERs. PM_2.5_ exposure promotes HIF1A expression by inducing IL-6 production in epithelial cells *in vitro*. HIF1A, a transcription factor necessary for the induction of the hypoxia response [75], has also been reported in lung mucosal biopsies from asthmatic patients, which could be related to tissue hypoxia in asthmatic airways [76]. Surprisingly, HIF1A was classified in the second module, probably due to its expression profile being closer to other transcription and growth factors in that module.

According to its module annotation, the hub gene PFKFB3 has been reported to be overexpressed in hypoxic conditions and encodes a metabolic enzyme that produces fructose-2,6-bisphosphate (F-2,6-BP), which promotes glycolysis. Thus, overexpression of PFKFB3 leads to abnormal glycolysis and compromises mitochondrial respiration [77].

These results suggest that PM_2.5_ exposure may contribute to the development or exacerbation of asthma by promoting Th2/Th17-mediated inflammation. The enrichment of biological processes associated with cytokine signaling, myeloid leukocyte migration, and response to cytokines further supports the role of immune dysregulation in linking PM_2.5_ exposure and asthma pathogenesis.

These findings have important clinical and public health implications. The common DEGs/GADERs identified and hub genes in preserved modules between co-expression networks could serve as potential biomarkers for monitoring the impact of air pollution exposure on asthma risk and severity. By understanding the molecular mechanisms underlying the link between PM_2.5_ and asthma, more targeted prevention strategies and personalized treatment approaches could be developed for individuals at risk of developing or experiencing asthma exacerbations due to air pollution exposure.

Previous transcriptomic studies investigating molecular signatures altered in asthma include a comprehensive analysis by Szczesny *et al*., which leveraged an extensive dataset of 583 samples to identify 389 asthma DEGs [78], and an eight microarray studies meta-analysis by Tsai *et al*. reporting 1273 asthma DEGs [79]. To evaluate the reproducibility of our findings, cross-comparisons were conducted between our results and those reported in these two studies. A total of 267 DEGs with similar expression patterns were replicated, representing 16% of the previously reported genes (Supplementary Fig. S5a and c).

These results align with expectations, as nearly 83% of the genes altered in asthma in our study were detected through epigenetic changes. This observation underscores the significance of identifying early transcriptional regulatory marks to gain deeper insights into the molecular mechanisms underlying the disease. When genes altered by PM_2.5_ exposure were incorporated into the cross-comparison, additional links between environmental exposure and disease pathogenesis were revealed (Supplementary Fig. S5b and d).

The selected datasets enabled the development of a bioinformatics approach capable of identifying common patterns at the transcriptomic and epigenomic levels in AECs, present both in asthma diagnosis and during PM_2.5_ exposure, which is a key finding that supports the need for public health measures. Specifically, we identified common DEGs, GADERs, and hub genes in preserved co-expression modules across conditions. By intersecting DEGs and GADERs, 349 common DEGs/GADERs that exhibited similar expression/regulation patterns were obtained. Additionally, the five most influential hub genes per module were identified based on their intramodular connectivity and can be prioritized for subsequent analyses and biological interpretation.

Future research directions should focus on longitudinal investigations to elucidate the temporal dynamics of the identified molecular signatures, as well as studies in animal models or clinical cohorts to establish the *in vivo* relevance and translational potential of these findings. Integrating additional data modalities, such as metabolomics or proteomics, could also provide a more holistic understanding of the complex interactions between environmental exposures, epigenetic regulation, and asthma development.

## Conclusion

The integrative analysis of transcriptomic and ChIP-seq data including network construction and analysis, allowed uncovering of shared transcriptomic and epigenomic signatures between PM_2.5_ exposure and asthma, highlighting potential molecular mechanisms that may underlie asthma development and exacerbation by PM_2.5_ exposure. Specifically, it was found that PM_2.5_ exposure in humans impairs epithelial migration and repair processes, hypoxia response, and the immune response, mainly through induction of IL-17 and TNF signaling pathways, and may lead to mitochondrial dysfunction, alterations which were also seen in the asthmatic datasets. These findings may contribute to guiding future research for the identification of potential biomarkers, the development of targeted interventions, and generating scientific evidence for the need to implement effective public health strategies to address the impact of air pollution on respiratory health.

## Materials and methods

### Dataset selection and acquisition

Datasets related to PM_2.5_ exposure and T2-high asthma transcriptomic and epigenomic data were obtained from the Gene Expression Omnibus (GEO) [9], and the Sequence Read Archive (SRA), both repositories hosted by the National Center for Biotechnology Information (NCBI). The datasets selected to identify PM_2.5_ exposure transcriptomic signatures are available with the codes GSE144770 [80], GSE175541 [81], and GSE243618 [82] on one hand; and on the other hand, the asthma diagnosis datasets are available in the GEO under accession codes GSE109313 [83], GSE175541 [81], and GSE144770 [80]. These datasets were specifically chosen based on their utilization of RNA-seq technologies to assess at least one of the conditions of interest in AECs. Similarly, datasets assessing epigenetic changes associated to PM_2.5_ exposure and asthma diagnosis are available with the codes SRP057970 in the SRA [7] and GSE109894 in the GEO [84], respectively; these datasets were specifically selected because they examined the same histone mark (H3K27ac) by ChIP-seq in cell types relevant to asthma pathophysiology. Table 1 provides a comprehensive characterization of these selected datasets along with their most relevant findings. A summarized schematic representation of the data analysis workflow is presented in Fig. 1.

### Transcriptomic raw data preprocessing

Each dataset was subjected to quality control using FastQC v0.12.0 [85] and MultiQC v1.16 [86]. Trimmomatic v0.39 [87] was used, if necessary, to remove adapters, as well as to filter sequences with an average Phred quality index less than 20 and length fewer than 50 bases. Due to the quality conditions of the reverse library from the GSE144770 dataset, only forward sequences were used in later steps, applying the same filters mentioned.

Transcriptomic datasets were mapped to the reference genome GRCh38.p14 by HISAT2 v2.2.1 [88], and the gene count matrices were obtained by the featureCounts function of the Subread package v2.0.2 [89]. In both cases, the source strand of the sequences was verified using the infer-experiment.py function of the RSeQC package [90].

### Epigenomic raw data preprocessing

Quality control from the obtained datasets was performed with FastQC [85] and MultiQC [86] tools. Fastp v.0.23.4 [91] was employed for the removal of low-quality reads (Phred quality score < 20). The resultant high-quality reads were mapped to the reference genome GRCh38.p14 with the Subread package v2.0.6 [92], followed by sorting, indexing and quality assessment steps with SAMTools v1.18 [93]. Later removal of PCR and optical duplicates was executed with Picard v3.1.1 [94]. Broad peak calling was conducted using MACS3 v3.0.0, applying an FDR threshold of 0.05 [95]. Finally, ChIP-seq quality assessment was performed through the R Bioconductor package ChIPQC v1.38.0 [96].

### Differential gene expression analysis

The count matrices obtained with featureCounts were subjected to quality control, in which those samples whose correlation with others from the same set was less than 0.8 were discarded, according to ENCODE recommendations for bulk RNA-seq [97]. Also, if necessary, the number of samples in treatment and control groups in each data set was balanced by random subsampling.

Subsequently, differential expression analysis was performed using the count matrices obtained from the quality control process using the likelihood ratio test or LRT, from the DESeq2 library [98, 99], available in R [100]. Genes with a *P*-value <.05 were considered as DEGs.

### H3K27ac differentially enriched regions analysis

The R Bioconductor package DiffBind v3.12.0 was employed to select a subset of confidence peaks, ensuring reproducibility and exclusion of blacklisted regions. Identification of H3K27ac Differentially enriched regions (DERs) in the asthma dataset was made using the DESeq2 method within DiffBind at α = 0.05 [98, 100, 101]. On the other hand, since the PM_2.5_ dataset showed low quality, among the subset of confidence peaks identified by Diffbind, DERs were considered as those absent in the other condition, replicating the method from its original publication [7]. Peak annotation was done using the nearest gene method through the R Bioconductor package ChIPseeker v1.38.0 [102]. Specifically, only peaks located in intergenic regions and those within a ±3kb window from the transcription start site (TSS), considered as promoter peaks were selected for subsequent analysis. To annotate differentially enriched enhancers, the peaks located within intergenic regions were intersected with the evidence-based location of enhancers from GeneHancer v5.18, using bedtools v2.31.1 [103, 104]. Given the presence of multiple enhancers associated with multiple genes, a subset of high confidence was selected based on their GeneHancer score and Gene-GeneHancer association score, considering only those surpassing their Q1 threshold (or P25) and having a maximum association of five genes per enhancer.

### Functional notation of common DEGs/GADERs

To elucidate the biological processes and molecular pathways linked with the common DEGs and shared GADERs in both conditions, ORA and GSEA were conducted. These analyses were performed using the clusterProfiler package [105, 106] on the Gene Ontology [107, 108] and KEGG databases [109, 110], as well as on the g:Profiler’s g:GOSt tool [111–113]. Visualizations of the functional enrichment results were generated using enrich plot v1.22 [114].

### Co-expression networks construction and analysis

First, for data preprocessing for the co-expression network construction and analysis, expression matrices from all RNA-seq datasets were merged by study group: Asthma Diagnosis and PM_2.5_ exposure. Later, to mitigate batch effects, the ComBat-seq method [115] from the sva library [116] was used. According to its developers, this method employs a negative binomial regression model, which retains the integer natures of the count data from RNA-seq throughout the batch adjustment process. A total of 37 samples were used for the construction of the asthma diagnosis network, and 18 for the PM_2.5_ exposure network. In the next step, genes with counts below 15 in more than half of the samples in their respective original dataset were filtered out from the batch-corrected count matrices. As the final step in the preprocessing phase, batch-corrected and filtered expression matrices were normalized with the variance stabilizing transformation [98]. Finally, expression of genes that were identified as differentially expressed or associated with DERs were subset.

After data preprocessing, the WGCNA algorithm was used to build two signed co-expression networks, one for each study group. During this process, the R BioNERO package was employed [117]. Pearson’s correlation coefficient was calculated for each pair of selected genes to construct correlation matrices. Subsequently, soft-thresholds were evaluated for the construction of the adjacency matrices to ensure that the resulting networks conformed to a scale-free topology with an *R*^2^ of approximately 0.8, thereby avoiding reduced mean connectivity. Consequently, asthma diagnosis co-expression network soft threshold was set at 12 and PM_2.5_ exposure at 17. Then, adjacency matrices were transformed into topological overlap matrices and further to topological overlap dissimilarity matrices. These matrices were used to detect modules through hierarchical clustering, with minimum module size of 30 genes and a height threshold of 0.25.

To assess the relationship between modules and conditions, module eigengenes, defined as the first principal component of the expression matrix, were correlated with the binarized conditions in each network. Modules with Pearson’s correlation *P*-value <.05 were considered as significantly correlated [71, 117]. Subsequently, module preservation analysis was conducted using the netRep algorithm with 1000 permutations. This algorithm employs nonparametric permutation analyzes to score preservation of modules from one network into the other [62].

Finally, hub genes were identified in significantly correlated and preserved modules by evaluating two conditions: genes in the 10% with highest degree and genes with module membership above 0.8; module membership defined as the Pearson’s correlation between expression profiles and module eigengene. Subsequently correlated and preserved module annotations were performed based on the most common terms in an enrichment analysis of modules genes over biological process terms in the Gene Ontology Database. In addition, hub genes were ranked according to their intramodular connectivity to select the top five hub genes with the highest connectivity for later analyses.

### Limitations of the study

This integration of transcriptomic and epigenomic data, while insightful, has several limitations. The use of public datasets introduces variability and potential biases due to differences in original experimental designs and sampling conditions, affecting reproducibility. The biological and functional implications derived from identified genes and pathways lack direct experimental validation. Uncontrolled external factors in the original studies, such as lifestyle and other pollutant exposures, may confound results and limit generalizability. The analysis does not consider temporal variability between acute and chronic responses to PM_2.5_ exposure and asthma. Furthermore, the focus on AECs excludes other potentially important cell types in PM_2.5_ response and asthma pathogenesis. While co-expression network analyses provide valuable insights, they cannot prove causality and are sensitive to certain parameters. These limitations highlight the need for a more comprehensive approach, including additional experimental validation, consideration of temporal dynamics, and controlled studies to better elucidate the molecular mechanisms linking PM_2.5_ exposure and asthma.

## Supplementary Material

dvae026_Supp

## Data Availability

Datasets included on this integrative omics analysis are publicly available on the GEO and the SRA repositories from the NCBI under the accession numbers GSE144770, GSE175541, GSE243618, GSE109313, GSE109894, and SRP057970.
