# Supplementary material for: Airborne fine particulate matter exposure induces transcriptomic alterations resembling asthmatic signatures: insights from integrated omics analysis
Source: Environ Epigenet. 2025 Jan 2;11(1):dvae026. doi: 10.1093/eep/dvae026 (PMC11753294; doi:10.1093/eep/dvae026)
Supplement: dvae026_Supp [file dvae026_supp.zip › suppl_data/Suppl. figures.docx]

**SUPPLEMENTARY FIGURES**

**
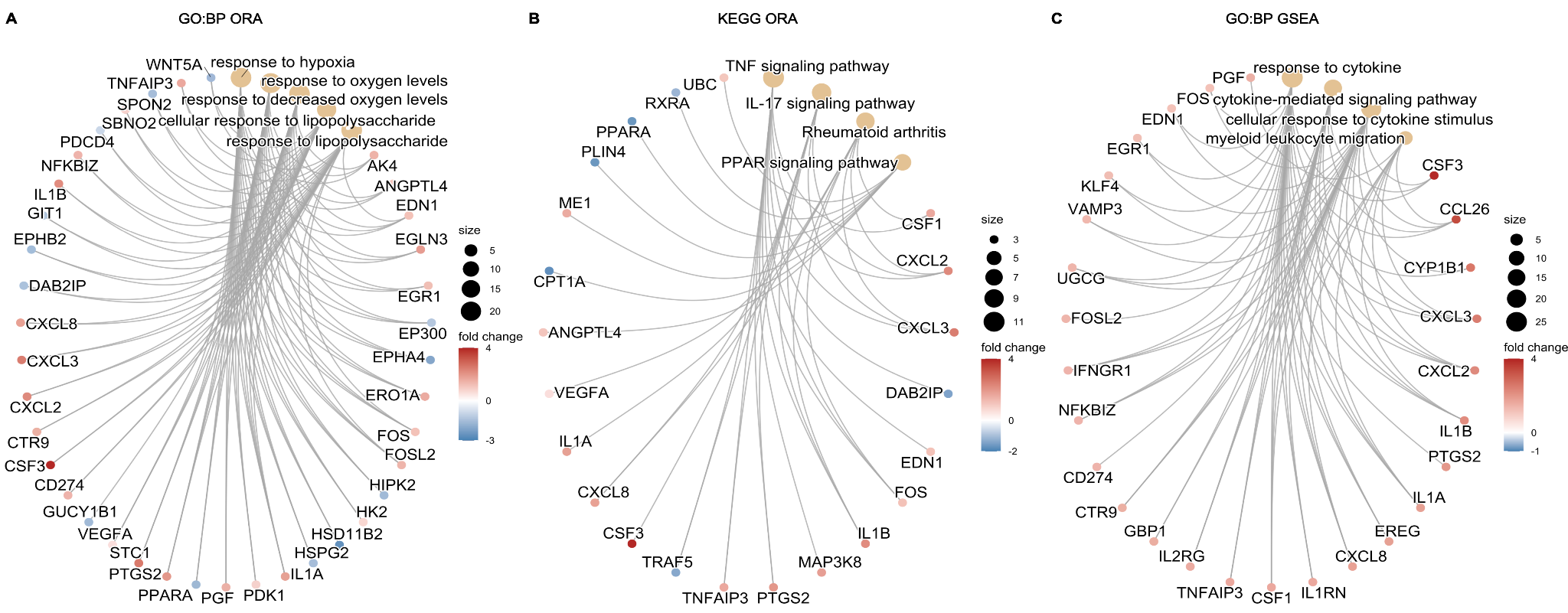
**

**Suppl. Figure 1. Gene-Concept networks of common DEGs/GADERs functional annotation.** Gene-Concept networks which depicts the linkages of genes to one or more of the enriched terms (yellow circles) identified through GO:BP ORA (A.), GSEA (C.), and pathways ORA (B.) of the 349 common DEGs/GADERs. The fold changes are represented as the mean log2FC calculated from both conditions.


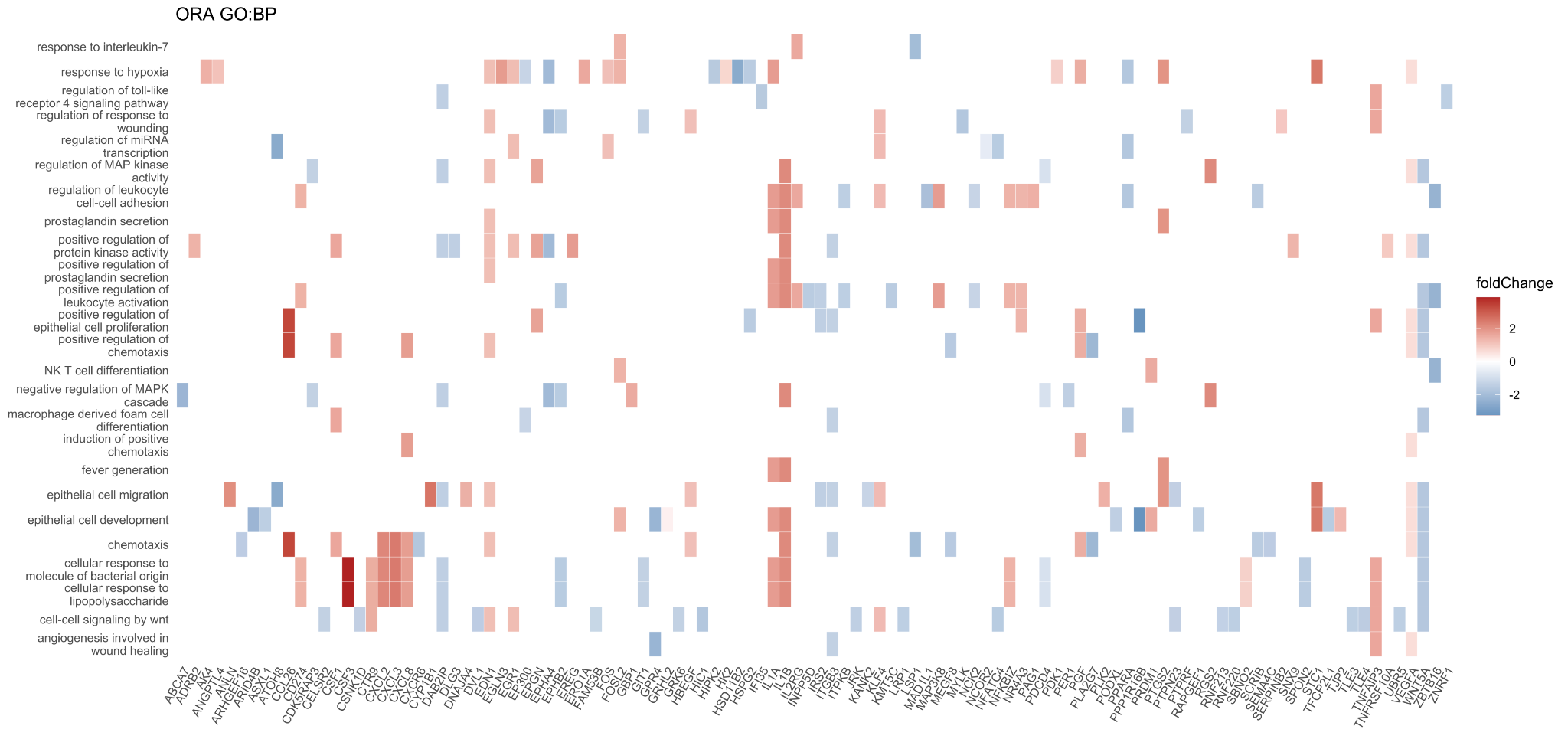


**Suppl. Figure 2. Main biological processes associated with common DEGs/GADERs.** Expression patterns heatplot of genes associated with 25 biological processes related to immune response, epithelium processes, and hypoxia response identified through ORA analysis of the 349 common DEGs/GADERs. The fold changes are represented as the mean log2FC calculated from both conditions.

**
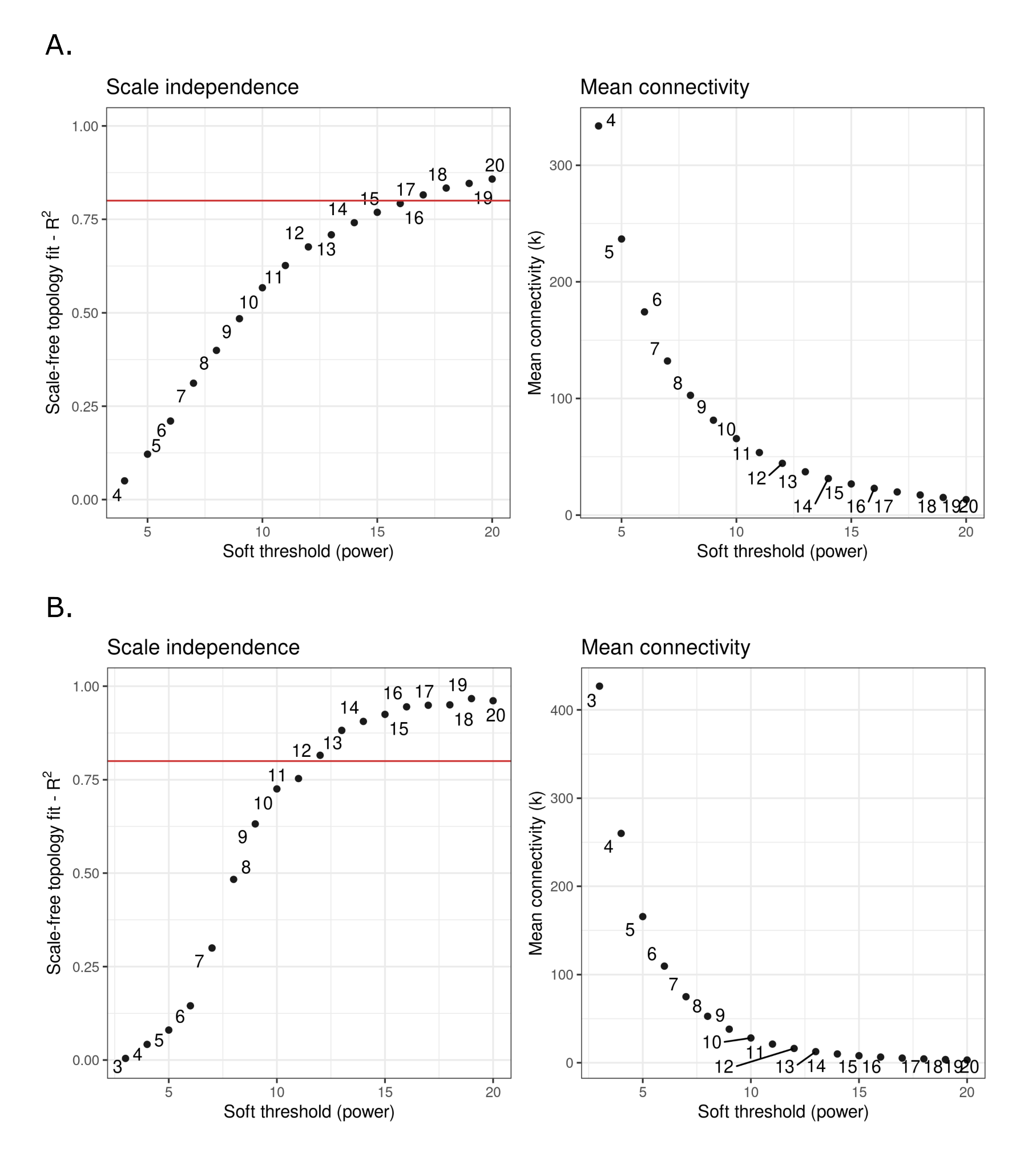
**

**Suppl. Figure 3.** **Scale independence and Mean connectivity plots.** Scale independence and mean connectivity plots used for soft threshold selection in (A.) PM_2.5_ exposure and (B.) asthma co-expression networks. In both plots thresholds are represented in the horizontal axis. In the Scale independence plots, the vertical axis represents the adjusted R^2^ to the scale-free topology model while in the Mean connectivity plots the vertical axis represents the mean connectivity for networks at a given threshold.


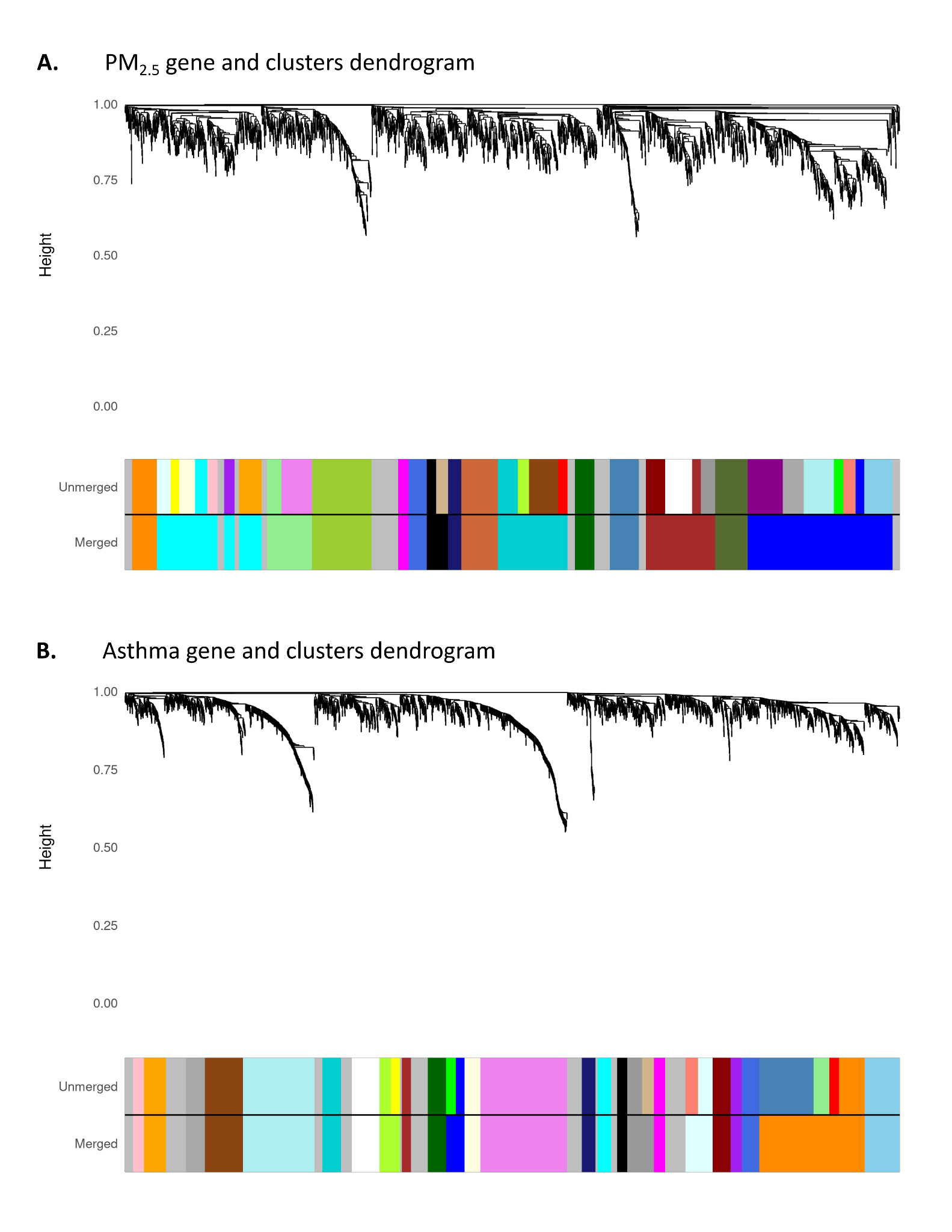


**Suppl. Figure 4. Hierarchical cluster dendrograms.** Hierarchical cluster dendrogram of (A.) PM_2.5_ exposure and (B.) asthma co-expression networks. Each branch represents a gene. The color rows below represent module membership by similarity before and after merging modules. Gray modules represent unsigned genes. After merging modules by similarity 25 and 18 co-expression modules were found in asthma and PM_2.5_ networks.


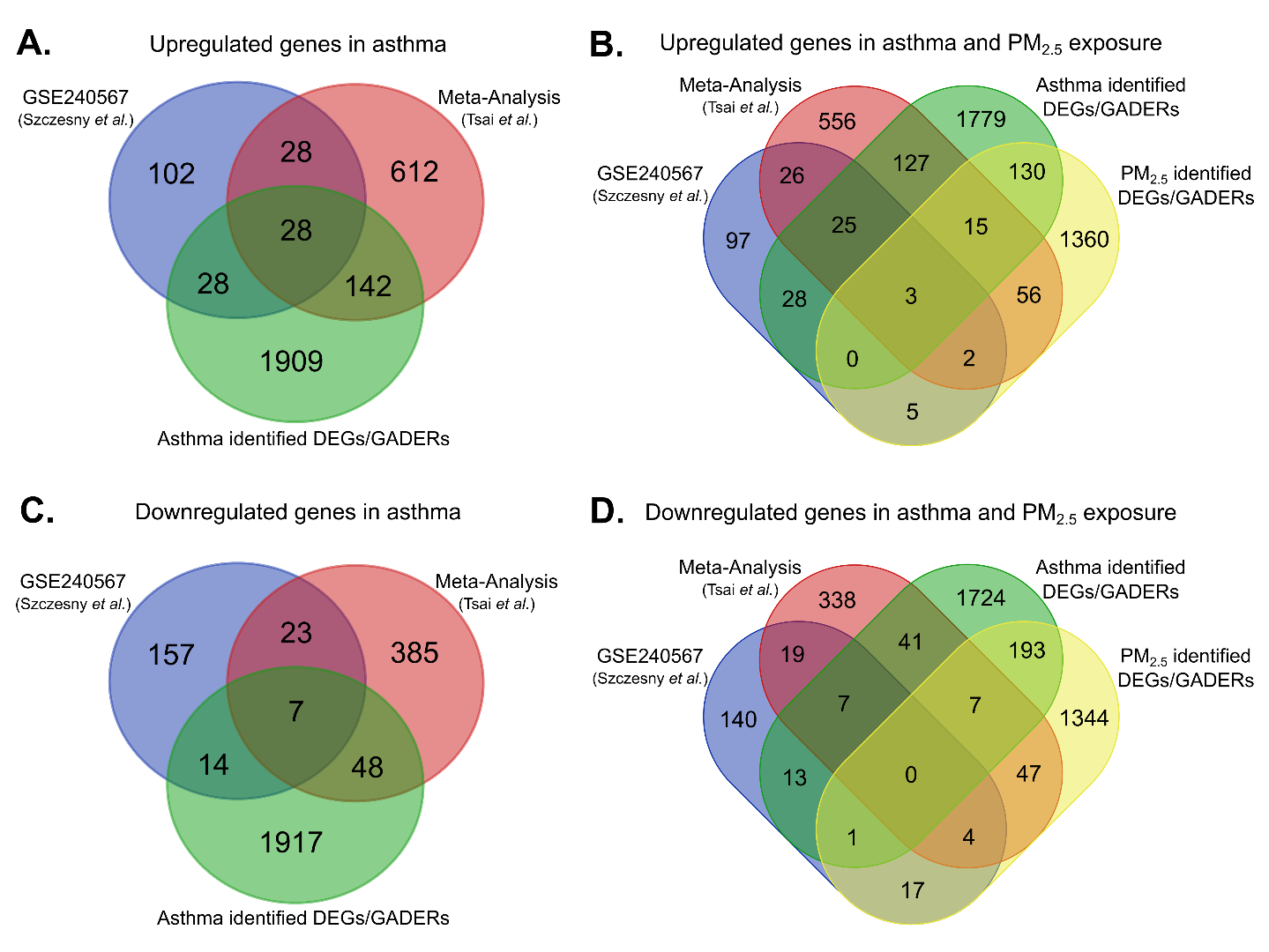


**Supplementary Figure 5. Cross-comparison of current findings with published literature.** Venn diagrams comparing the altered genes identified in our study with those reported in two previously published studies. The diagrams show: (A.) Overlap of upregulated genes in asthma between our findings (green) and published studies, (B.) Comparison between all asthma-upregulated genes and our PM2.5 exposure findings, (C.) Overlap of downregulated genes in asthma between our findings (green) and published studies, and (D.) Comparison between all asthma-downregulated genes and our PM2.5 exposure findings.
