# Supplementary material for: Airborne fine particulate matter exposure induces transcriptomic alterations resembling asthmatic signatures: insights from integrated omics analysis
Source: Environ Epigenet. 2025 Jan 2;11(1):dvae026. doi: 10.1093/eep/dvae026 (PMC11753294; doi:10.1093/eep/dvae026)
Supplement: dvae026_Supp [file dvae026_supp.zip › suppl_data/SupplTable1.pdf]

Supplementary Table 1. Complete list of common differential expressed genes discovered between PM2.5 exposure and asthma diagnosis. The fold change is depicted as mean log2FC from datasets. (Attached in Suppl. tables.xlsx; 63,1 KB)

geneID	lfc_RNA-seq_PM2.5	H3K27ac_status_PM2.5	lfc_ChIP-seq_PM2.5	lfc_RNA-seq_asthma	H3K27ac_status_asthma	lfc_ChIP-seq_asthma
A4GALT	-0,37007911282536	NA	NA	NA	DECREASED_H3K27AC_IN_PROMOTER_AND_ENHANCER	-1,87777644208347
AAAS	NA	INCREASED_H3K27AC_IN_PROMOTER	1,68735878823563	NA	INCREASED_H3K27AC_IN_ENHANCER	2,39379213294743
ABCA7	NA	DECREASED_H3K27AC_IN_PROMOTER	-1,93511357660106	NA	DECREASED_H3K27AC_IN_PROMOTER	-2,43072484832528
ABTB2	0,490898052797565	NA	NA	NA	INCREASED_H3K27AC_IN_ENHANCER	2,4773497467461
ACAT2	0,856435545880835	NA	NA	NA	INCREASED_H3K27AC_IN_PROMOTER	2,31490552068729
ACTN1	NA	DECREASED_H3K27AC_IN_PROMOTER	-1,93611482787209	NA	DECREASED_H3K27AC_IN_PROMOTER	-2,50528435715818
ADAMTS1	0,855906652794202	NA	NA	2,06660209177707	NA	NA
ADGRF1	0,473625333713756	NA	NA	NA	INCREASED_H3K27AC_IN_PROMOTER_AND_ENHANCER	2,26593805296119
ADRB2	0,562137458650028	NA	NA	NA	INCREASED_H3K27AC_IN_ENHANCER	2,25069093560028
AFAP1	-0,40817401900276	NA	NA	NA	DECREASED_H3K27AC_IN_PROMOTER	-2,21132794913609
AK4	0,362741085434613	NA	NA	NA	INCREASED_H3K27AC_IN_ENHANCER	2,47124698960329
ALDH1A3	0,780988344534974	NA	NA	0,901293939776653	NA	NA
ANGPTL4	0,597084539290257	NA	NA	1,57303399042041	NA	NA
ANKRA2	-0,51137546028456	NA	NA	-0,739344047548367	NA	NA
ANKRD65	-0,45544443112167	NA	NA	NA	DECREASED_H3K27AC_IN_PROMOTER	-1,29744822211262
ANLN	1,64181255825648	NA	NA	NA	INCREASED_H3K27AC_IN_PROMOTER	2,50016940083339
ANO7	NA	INCREASED_H3K27AC_IN_PROMOTER	1,68316538470952	0,906225287653197	NA	NA
ARFGAP3	-0,42850640075576	NA	NA	NA	DECREASED_H3K27AC_IN_ENHANCER	0,0684136621302783
ARHGEF10L	-0,48748002192938	NA	NA	NA	DECREASED_H3K27AC_IN_PROMOTER	-2,27795994833056
ARHGEF16	-0,32478418736244	NA	NA	NA	DECREASED_H3K27AC_IN_PROMOTER	-2,61927590104176
ARHGEF17	-0,33780614434284	NA	NA	NA	DECREASED_H3K27AC_IN_PROMOTER	-2,46103265620263
ARID4B	NA	DECREASED_H3K27AC_IN_ENHANCER	-1,91383312110074	NA	DECREASED_H3K27AC_IN_ENHANCER	-2,43902524065161
ARL4C	-0,32984060418355	NA	NA	NA	DECREASED_H3K27AC_IN_ENHANCER	-2,40845644584488
ARPIN	NA	DECREASED_H3K27AC_IN_ENHANCER	-1,83392554808091	NA	DECREASED_H3K27AC_IN_PROMOTER	-2,25429142616332
ASAP3	-0,69672756935474	NA	NA	NA	DECREASED_H3K27AC_IN_PROMOTER	-2,43462046407014
ASB13	-0,51663395539329	NA	NA	NA	DECREASED_H3K27AC_IN_ENHANCER	-2,51373767198859
ASXL1	-0,35263461239303	NA	NA	NA	DECREASED_H3K27AC_IN_PROMOTER	-2,54007660252624
ATOH8	-2,82085925559223	NA	NA	NA	DECREASED_H3K27AC_IN_ENHANCER	-2,30261164856582
ATP10A	-1,47551698050325	NA	NA	NA	DECREASED_H3K27AC_IN_PROMOTER	-2,35315531510371
B3GNT7	-0,5316212275268	NA	NA	NA	DECREASED_H3K27AC_IN_PROMOTER	-2,16907722388829
BOD1	NA	DECREASED_H3K27AC_IN_ENHANCER	-1,63635912003097	NA	DECREASED_H3K27AC_IN_ENHANCER	-2,35649104635188
CA2	0,92955122726643	NA	NA	2,58156347091752	NA	NA
CA8	-1,58517691240667	NA	NA	-0,853340100967397	NA	NA
CAMSAP1	-0,32871945699805	NA	NA	NA	DECREASED_H3K27AC_IN_PROMOTER	-2,5533262804523
CAPZB	NA	DECREASED_H3K27AC_IN_PROMOTER	-0,938216340231293	NA	DECREASED_H3K27AC_IN_PROMOTER	-2,31164930546221
CCDC57	NA	DECREASED_H3K27AC_IN_PROMOTER	-1,64944295927078	NA	DECREASED_H3K27AC_IN_PROMOTER	-2,20227047885456
CCDC80	-0,6930448168485	NA	NA	-0,689862097371371	NA	NA
CCDC85C	-0,457885785708 37	NA	NA	NA	DECREASED_H3K27AC_IN_PROMOTER	-2,57655051534969
CCL26	2,21147405773726	NA	NA	4,51287703063192	NA	NA
CD274	0,566652448346674	NA	NA	NA	INCREASED_H3K27AC_IN_PROMOTER	2,25849082662071
CDC42BPB	-0,26643011131793	NA	NA	NA	DECREASED_H3K27AC_IN_PROMOTER	-2,38327863888601
CDC42EP5	0,532782497127066	NA	NA	0,790501981301944	NA	NA
CDK5RAP2	0,296808635953994	NA	NA	NA	INCREASED_H3K27AC_IN_PROMOTER	2,31094557144477
CDK5RAP3	-0,41991320635857	NA	NA	NA	DECREASED_H3K27AC_IN_ENHANCER	-2,29692862721431
CDX1	-1,71884439557284	NA	NA	NA	DECREASED_H3K27AC_IN_ENHANCER	-2,64969997526906
CELSR2	-0,37388911024863	NA	NA	NA	DECREASED_H3K27AC_IN_PROMOTER	-2,34036368177142
CERK	-0,65906155891484	NA	NA	NA	DECREASED_H3K27AC_IN_PROMOTER	-2,53940842645976
CLDN8	-1,63113074374124	NA	NA	-1,80132182543878	NA	NA
CLEC2B	1,630299506069	NA	NA	NA	INCREASED_H3K27AC_IN_PROMOTER	2,7104686963888
CLN8	-0,36129270021817	NA	NA	NA	DECREASED_H3K27AC_IN_ENHANCER	-2,33695388261937
CLUH	-0,3610715463438	NA	NA	NA	DECREASED_H3K27AC_IN_PROMOTER	-2,48619488409751
CPT1A	-0,84807442539913	NA	NA	NA	DECREASED_H3K27AC_IN_PROMOTER	-2,6386305945535
CRACD	-0,99910694660846	NA	NA	NA	DECREASED_H3K27AC_IN_PROMOTER	-2,59840872822476
CRACDL	-0,76369160693971	NA	NA	NA	DECREASED_H3K27AC_IN_PROMOTER	-1,6373792225843
CSF1	0,882734498271256	NA	NA	NA	INCREASED_H3K27AC_IN_ENHANCER	2,33257113482587
CSF3	4,73891770938215	NA	NA	3,05658905862644	NA	NA

CSNK1D	-0,28438093548949	NA	NA	NA	DECREASED_H3K27AC_IN_ENHANCER	-2,44535043325214
CTR9	0,376445003964892	NA	NA	NA	INCREASED_H3K27AC_IN_ENHANCER	2,63253191269647
CXCL2	2,00988416418534	NA	NA	NA	INCREASED_H3K27AC_IN_ENHANCER	2,38677267574114
CXCL3	2,48054044441233	NA	NA	NA	INCREASED_H3K27AC_IN_ENHANCER	2,38677267574114
CXCL8	1,15566070315321	NA	NA	NA	INCREASED_H3K27AC_IN_ENHANCER	2,38810572847408
CXCR6	NA	DECREASED_H3K27AC_IN_ENHANCER	-1,92848601429835	-1,38084000329668	NA	NA
CYP1B1	3,61099894037417	NA	NA	NA	INCREASED_H3K27AC_IN_PROMOTER	1,48479904415209
CYP2J2	0,322054284920592	NA	NA	NA	INCREASED_H3K27AC_IN_ENHANCER	2,30043054683879
CYP4B1	-0,37678813505441	NA	NA	NA	DECREASED_H3K27AC_IN_ENHANCER	-2,46637556042811
DAB2IP	-0,3519530641555	NA	NA	NA	DECREASED_H3K27AC_IN_PROMOTER	-2,46604612184978
DDX47	1,14597921570439	NA	NA	NA	INCREASED_H3K27AC_IN_ENHANCER	2,42811949275745
DENND3	-0,44778299440211	NA	NA	NA	DECREASED_H3K27AC_IN_PROMOTER_AND_ENHANCER	-2,48929972602354
DEPTOR	-1,2104376413456	NA	NA	-1,76947432422428	NA	NA
DGLUCY	-0,77253722842143	NA	NA	-0,335313645335253	NA	NA
DLG3	-0,38036055455191	NA	NA	NA	DECREASED_H3K27AC_IN_PROMOTER	-2,36358959618645
DLGAP4	NA	INCREASED_H3K27AC_IN_PROMOTER	2,07587456174277	0,676131931569473	NA	NA
DNAJA4	0,660903533321474	NA	NA	NA	INCREASED_H3K27AC_IN_ENHANCER	2,24624919755848
DNAJB11	0,398788573225866	NA	NA	0,256299573384573	NA	NA
DNAJC8	0,287925751633087	NA	NA	NA	INCREASED_H3K27AC_IN_ENHANCER	2,21689177971274
DSP-AS1	0,986726053631586	NA	NA	NA	INCREASED_H3K27AC_IN_PROMOTER	2,22468984114723
DVL1	-0,32724520779148	NA	NA	NA	DECREASED_H3K27AC_IN_PROMOTER	-2,6084301230854
EDN1	0,671701181611117	NA	NA	1,65012256090639	NA	NA
EGLN3	2,96777676891577	NA	NA	0,705636502717911	NA	NA
EGR1	1,30531070540309	NA	NA	1,09107817026628	NA	NA
EHD1	0,4093487428235	NA	NA	1,38241118336897	NA	NA
ELK4	-0,46512794659572	NA	NA	NA	DECREASED_H3K27AC_IN_PROMOTER	-2,30001153637014
ENSG00000174171	-0,94225580107791	NA	NA	-0,038091443960973	NA	NA
ENTPD4	-0,3163629049548	NA	NA	NA	DECREASED_H3K27AC_IN_PROMOTER	-2,39739814193225
EP300	-0,34580390624199	NA	NA	NA	DECREASED_H3K27AC_IN_ENHANCER	-2,1654655157517
EPGN	1,17000176588415	NA	NA	NA	INCREASED_H3K27AC_IN_PROMOTER	2,21298376805028
EPHA4	-1,76806358646805	NA	NA	NA	DECREASED_H3K27AC_IN_PROMOTER	-2,37214786779183
EPHB2	-0,65149245791772	NA	NA	NA	DECREASED_H3K27AC_IN_PROMOTER	-2,40789447239175
EREG	1,15336951564287	NA	NA	NA	INCREASED_H3K27AC_IN_ENHANCER	2,42508996174301
ERICH5	-0,4071347697867	NA	NA	-1,48817948004291	NA	NA
ERO1A	0,641741495125075	NA	NA	NA	INCREASED_H3K27AC_IN_PROMOTER_AND_ENHANCER	2,49628408723585
ESPN	NA	DECREASED_H3K27AC_IN_PROMOTER	-1,89971928070128	NA	DECREASED_H3K27AC_IN_PROMOTER	-2,29029300073266
FAIM	0,73591596093614	NA	NA	NA	INCREASED_H3K27AC_IN_ENHANCER	2,20523064376433
FAM110C	0,508310593833814	NA	NA	1,03068777885024	NA	NA
FAM133B	0,520727971910059	NA	NA	NA	INCREASED_H3K27AC_IN_ENHANCER	2,52581065512089
FAM3C	NA	INCREASED_H3K27AC_IN_ENHANCER	0,996507192702794	NA	INCREASED_H3K27AC_IN_ENHANCER	2,28856510863148
FAM53B	-0,58909376413373	NA	NA	NA	DECREASED_H3K27AC_IN_ENHANCER	-1,81571127291082
FAM78A	NA	DECREASED_H3K27AC_IN_PROMOTER	-2,02696138204695	NA	DECREASED_H3K27AC_IN_PROMOTER	-1,89769240877973
FBXL14	0,855813807097826	NA	NA	0,771164524035221	NA	NA
FBXO17	-0,81766085604481	NA	NA	NA	DECREASED_H3K27AC_IN_PROMOTER	-2,5473580470421
FDFT1	0,731582384081831	NA	NA	NA	INCREASED_H3K27AC_IN_PROMOTER	2,44196278379768
FKBP5	-0,67920200521956	NA	NA	-2,5713776301913	NA	NA
FLNC	NA	INCREASED_H3K27AC_IN_PROMOTER	2,20951026988367	2,18674987449905	NA	NA
FNDC4	0,512328028788678	NA	NA	NA	INCREASED_H3K27AC_IN_ENHANCER	2,31159919838516
FOS	0,975238670768193	NA	NA	1,33163054757048	NA	NA
FOSL2	0,386673098108441	NA	NA	NA	INCREASED_H3K27AC_IN_ENHANCER	2,39625209033552
FRMD4A	-0,8237371243598	NA	NA	NA	DECREASED_H3K27AC_IN_PROMOTER	-2,43307553310852
GBE1	0,34018580981348	NA	NA	NA	INCREASED_H3K27AC_IN_PROMOTER	2,48976381438883
GBP1	0,679763627523915	NA	NA	NA	INCREASED_H3K27AC_IN_PROMOTER	2,39950667958857
GGA3	-0,29308107595834	NA	NA	NA	DECREASED_H3K27AC_IN_PROMOTER	-2,46039599734363
GGT5	NA	INCREASED_H3K27AC_IN_PROMOTER_AND_ENHANCER	0,996507192702794	3,20992204014847	NA	NA
GIT1	-0,29823742853243	NA	NA	NA	DECREASED_H3K27AC_IN_PROMOTER	-2,36998583103007
GOLGA3	-0,36322373113104	NA	NA	NA	DECREASED_H3K27AC_IN_ENHANCER	-2,20135518373191
GON4L	NA	INCREASED_H3K27AC_IN_ENHANCER	2,13995246780744	NA	INCREASED_H3K27AC_IN_PROMOTER	2,84212908083625
GPALPP1	0,450563506132812	NA	NA	NA	INCREASED_H3K27AC_IN_ENHANCER	2,13728414260885

GPR4	NA	DECREASED_H3K27AC_IN_PROMOTER	-1,93611482787209	NA	DECREASED_H3K27AC_IN_PROMOTER	-2,49690183999189
GRAMD1A	-0,50439296223738	NA	NA	NA	DECREASED_H3K27AC_IN_PROMOTER	-2,53781552726883
GRAMD2A	-0,77559385514208	NA	NA	NA	DECREASED_H3K27AC_IN_PROMOTER	-2,4183285790486
GRAMD4	-0,55532356677311	NA	NA	NA	DECREASED_H3K27AC_IN_PROMOTER	-2,56140141580389
GRHL2	-0,62648197060108	NA	NA	NA	DECREASED_H3K27AC_IN_ENHANCER	1,1023541132886
GRK6	-0,38159614857247	NA	NA	NA	DECREASED_H3K27AC_IN_PROMOTER	-2,2779383113817
GTF2A2	0,350800066251684	NA	NA	NA	INCREASED_H3K27AC_IN_ENHANCER	2,17097079631234
GUCY1B1	-0,39864945891209	NA	NA	NA	DECREASED_H3K27AC_IN_PROMOTER	-2,75617892089383
HBEGF	1,11208180497523	NA	NA	1,18149235305964	NA	NA
HIC1	-0,90482374918657	NA	NA	NA	DECREASED_H3K27AC_IN_PROMOTER	-2,42240188992522
HIPK2	-0,55238306698312	NA	NA	NA	DECREASED_H3K27AC_IN_PROMOTER	-2,62430437001754
HK2	0,373819385295527	NA	NA	1,107933988485	NA	NA
HSD11B2	-0,7624326874596	NA	NA	-4,37198177224004	NA	NA
HSPA7	6,95248964401691	NA	NA	NA	INCREASED_H3K27AC_IN_ENHANCER	2,47500236911741
HSPG2	-0,70295030148907	NA	NA	NA	DECREASED_H3K27AC_IN_PROMOTER	-2,33036888655431
IER3	1,12124281348313	NA	NA	0,424319270597877	NA	NA
IFI35	-0,65926384571927	NA	NA	NA	DECREASED_H3K27AC_IN_PROMOTER	-2,51680946145713
IFNGR1	0,321549469511545	NA	NA	NA	INCREASED_H3K27AC_IN_ENHANCER	2,48849994482017
IFRD1	0,454564855220239	NA	NA	NA	INCREASED_H3K27AC_IN_PROMOTER	2,34746569183632
IFT81	NA	INCREASED_H3K27AC_IN_PROMOTER	1,61702630891981	NA	INCREASED_H3K27AC_IN_ENHANCER	2,2844910588946
IGFBP3	0,386981982849009	NA	NA	NA	INCREASED_H3K27AC_IN_ENHANCER	2,29626368682077
IL1A	1,32168389163683	NA	NA	1,68339946416869	INCREASED_H3K27AC_IN_ENHANCER	2,38304261736184
IL1B	1,95706560156267	NA	NA	NA	INCREASED_H3K27AC_IN_ENHANCER	2,38304261736184
IL1RN	NA	INCREASED_H3K27AC_IN_PROMOTER	0,859415861373113	NA	INCREASED_H3K27AC_IN_ENHANCER	2,38304261736184
IL2RG	1,5984799322978	NA	NA	1,5397587987478	NA	NA
ING2	0,812373082961158	NA	NA	NA	INCREASED_H3K27AC_IN_ENHANCER	2,32528868248596
INPP5D	-0,60624612367171	NA	NA	NA	DECREASED_H3K27AC_IN_PROMOTER	-2,35661165950134
IRS2	-0,39851040353729	NA	NA	NA	DECREASED_H3K27AC_IN_ENHANCER	-2,42230823873999
ITGB3	-0,83246826473401	NA	NA	NA	DECREASED_H3K27AC_IN_PROMOTER	-1,8880424384874
ITGB4	-0,54894830373274	NA	NA	NA	DECREASED_H3K27AC_IN_PROMOTER	-2,64617929426842
ITPKB	-0,43370122125673	NA	NA	NA	DECREASED_H3K27AC_IN_PROMOTER	-2,45664624872676
ITPRIPL2	-0,80631819785693	NA	NA	NA	DECREASED_H3K27AC_IN_PROMOTER	-2,42859361019026
JADE2	-0,54571409864299	NA	NA	NA	DECREASED_H3K27AC_IN_PROMOTER	-2,17894746731705
JRK	-0,67790474882915	NA	NA	NA	DECREASED_H3K27AC_IN_PROMOTER	-2,13699041964713
KANK2	-0,52443108794538	NA	NA	NA	DECREASED_H3K27AC_IN_PROMOTER	-1,9063960518806
KIAA0513	-0,80600888365054	NA	NA	NA	DECREASED_H3K27AC_IN_PROMOTER	-2,40039030453689
KLF13	-0,36797811276412	NA	NA	NA	DECREASED_H3K27AC_IN_ENHANCER	-2,5224970222883
KLF16	-0,45847098027706	NA	NA	NA	DECREASED_H3K27AC_IN_ENHANCER	-1,75827355642352
KLF4	0,70241609762971	NA	NA	0,737089466476974	INCREASED_H3K27AC_IN_ENHANCER	2,26216136592871
KLHL29	-0,51736182033491	NA	NA	NA	DECREASED_H3K27AC_IN_PROMOTER	-2,53305984830225
KLHL36	-0,4021734626248	NA	NA	NA	DECREASED_H3K27AC_IN_ENHANCER	-2,31603928370102
KMT2D	-0,40864127421426	NA	NA	NA	DECREASED_H3K27AC_IN_PROMOTER	-2,58316026744577
KMT5C	-0,47384787601721	NA	NA	NA	DECREASED_H3K27AC_IN_PROMOTER	-2,5589461466782
KRT15	-0,37460963203173	NA	NA	-1,56150012905609	NA	NA
LAMB3	0,379121997837385	NA	NA	1,02731199339039	NA	NA
LFNG	-1,47041056627895	NA	NA	NA	DECREASED_H3K27AC_IN_PROMOTER	-2,47950188203568
LINC00342	0,182688644261005	NA	NA	1,44711085883466	NA	NA
LINC00880	1,687170185861	NA	NA	NA	INCREASED_H3K27AC_IN_PROMOTER	2,22076451895021
LIPG	1,53043142201227	NA	NA	2,73190850904554	NA	NA
LMBR1	-0,31339354685579	NA	NA	NA	DECREASED_H3K27AC_IN_PROMOTER	-2,54147658972159
LRP1	-0,33084584185149	NA	NA	NA	DECREASED_H3K27AC_IN_PROMOTER	-2,49918219230451
LRRC4	-0,71963985390366	NA	NA	NA	DECREASED_H3K27AC_IN_ENHANCER	1,28170463938387
LRRC49	0,803973954398819	NA	NA	NA	INCREASED_H3K27AC_IN_ENHANCER	2,47457163204957
LSP1	NA	DECREASED_H3K27AC_IN_ENHANCER	-1,38363441702782	NA	DECREASED_H3K27AC_IN_PROMOTER	-2,6252765156123
LTBP4	NA	DECREASED_H3K27AC_IN_PROMOTER	-1,18822862203468	NA	DECREASED_H3K27AC_IN_PROMOTER	-2,58733201796847
MAD1L1	NA	DECREASED_H3K27AC_IN_PROMOTER	-1,28139020077398	NA	DECREASED_H3K27AC_IN_PROMOTER	-2,46656133084008
MANSC1	-0,50745976820923	NA	NA	NA	DECREASED_H3K27AC_IN_PROMOTER	-2,16455508864675
MAP3K8	1,09940691172945	NA	NA	NA	INCREASED_H3K27AC_IN_ENHANCER	2,50755859836037
ME1	0,875295226261604	NA	NA	NA	INCREASED_H3K27AC_IN_PROMOTER	2,27753929927582

MEGF8	-0,77827286385763	NA	NA	NA	DECREASED_H3K27AC_IN_PROMOTER	-2,35836024245297
METTL27	NA	DECREASED_H3K27AC_IN_ENHANCER	-1,64944295927078	NA	DECREASED_H3K27AC_IN_ENHANCER	-2,16047641413146
MIGA1	-0,3429155701188	NA	NA	NA	DECREASED_H3K27AC_IN_ENHANCER	-2,24814457492604
MIR210HG	1,25627808448468	NA	NA	0,642375143002873	NA	NA
MIR6889	NA	DECREASED_H3K27AC_IN_PROMOTER	-1,89247198528727	NA	DECREASED_H3K27AC_IN_PROMOTER	-2,50528548461482
MIRLET7BHG	-0,58809418058623	NA	NA	NA	DECREASED_H3K27AC_IN_PROMOTER	-1,19570038138642
MLH3	-0,58500107121888	NA	NA	NA	DECREASED_H3K27AC_IN_ENHANCER	-2,1787426993795
MLLT6	-0,2791403129296	NA	NA	NA	DECREASED_H3K27AC_IN_PROMOTER_AND_ENHANCER	-2,55487160366944
MN1	-0,58995097675648	NA	NA	NA	DECREASED_H3K27AC_IN_PROMOTER	-2,60564939249122
MORC4	0,331496971589187	NA	NA	NA	INCREASED_H3K27AC_IN_ENHANCER	2,23850614168604
MRPS27	0,339424931526073	NA	NA	NA	INCREASED_H3K27AC_IN_PROMOTER	2,13440521938127
MT-ND2	-1,06570461233879	NA	NA	-0,487630613797052	NA	NA
MT-ND3	-0,91260209243723	NA	NA	-0,855745519288705	NA	NA
MT-ND4L	-0,89781615624982	NA	NA	-0,529126309285212	NA	NA
MTFR1	-0,35693993814623	NA	NA	NA	DECREASED_H3K27AC_IN_ENHANCER	-2,25143597518626
MTND4P12	-0,88136084382525	NA	NA	-1,26852857156177	NA	NA
MUC20P1	-0,55152126083991	NA	NA	NA	DECREASED_H3K27AC_IN_ENHANCER	-2,21701625667469
MUC4	-0,40455515795168	NA	NA	NA	DECREASED_H3K27AC_IN_ENHANCER	-2,21701625667469
MYLK	-0,96256595139814	NA	NA	NA	DECREASED_H3K27AC_IN_PROMOTER	-2,35495158095175
MYO18A	-0,55406933717149	NA	NA	NA	DECREASED_H3K27AC_IN_PROMOTER	-2,37361852115118
MYRF	-0,93439864248838	NA	NA	NA	DECREASED_H3K27AC_IN_PROMOTER	-2,65413763619313
NCK2	-0,40713080936033	NA	NA	NA	DECREASED_H3K27AC_IN_PROMOTER	-2,10031161951568
NCOA4	0,206481200094638	NA	NA	NA	INCREASED_H3K27AC_IN_PROMOTER	2,45584285663664
NCOR2	-0,5700168764004	NA	NA	NA	DECREASED_H3K27AC_IN_PROMOTER_AND_ENHANCER	-0,465626587967963
NEURL1B	-0,57296883326164	NA	NA	NA	DECREASED_H3K27AC_IN_PROMOTER	-2,68147804226913
NFATC4	-0,53116053504027	NA	NA	NA	DECREASED_H3K27AC_IN_PROMOTER	-2,51728613218369
NFIL3	0,614594663582359	NA	NA	0,759637266397022	NA	NA
NFKBIZ	0,958390573110446	NA	NA	NA	INCREASED_H3K27AC_IN_ENHANCER	1,86193400605302
NINL	-0,56804270461232	NA	NA	NA	DECREASED_H3K27AC_IN_PROMOTER	-2,23218661100068
NR4A3	1,71588899428701	NA	NA	0,973844407388352	NA	NA
NRG2	-1,55500557373676	NA	NA	NA	DECREASED_H3K27AC_IN_PROMOTER	-2,44145415705935
NT5DC2	-0,47153747304658	NA	NA	NA	DECREASED_H3K27AC_IN_PROMOTER	-2,34575081553371
NUP58	0,410888873968828	NA	NA	NA	INCREASED_H3K27AC_IN_PROMOTER	2,30869212658442
OBSCN	-0,63935728577373	NA	NA	NA	DECREASED_H3K27AC_IN_PROMOTER	-2,51169919761655
OCA2	1,9111554067916	NA	NA	NA	INCREASED_H3K27AC_IN_PROMOTER	2,31082229372584
PAG1	0,566270420194118	NA	NA	NA	INCREASED_H3K27AC_IN_ENHANCER	2,29627876162624
PARN	0,377015173569563	INCREASED_H3K27AC_IN_PROMOTER	2,23632063836729	NA	INCREASED_H3K27AC_IN_ENHANCER	1,46467007940659
PCDHGC3	-0,47150961682742	NA	NA	NA	DECREASED_H3K27AC_IN_ENHANCER	-2,49892151690736
PCSK6	NA	INCREASED_H3K27AC_IN_PROMOTER	1,92004421315404	1,68719847585347	NA	NA
PDCD4	-0,92877663333755	NA	NA	-0,853385723949603	NA	NA
PDK1	0,813052519241384	NA	NA	0,861273406861759	NA	NA
PDLIM4	NA	INCREASED_H3K27AC_IN_PROMOTER	1,95145756077416	1,24356956561086	NA	NA
PDP1	0,483601465016151	NA	NA	NA	INCREASED_H3K27AC_IN_ENHANCER	2,27523505207739
PER1	-0,45612308685482	NA	NA	NA	DECREASED_H3K27AC_IN_PROMOTER	-2,34936882654925
PGAP6	NA	DECREASED_H3K27AC_IN_PROMOTER	-0,760234005995989	NA	DECREASED_H3K27AC_IN_PROMOTER	-2,49625259498521
PGF	0,821754968447403	NA	NA	2,16196527613037	NA	NA
PGM5	NA	DECREASED_H3K27AC_IN_PROMOTER	-1,4901811919319	-1,13207991503052	NA	NA
PHLDA2	0,505092080995703	NA	NA	1,20796113163045	NA	NA
PHLDB1	-0,73912860273728	NA	NA	NA	DECREASED_H3K27AC_IN_PROMOTER	-2,3434500743212
PITPNM2	-0,4078323264446	NA	NA	NA	DECREASED_H3K27AC_IN_PROMOTER	-2,53811462428617
PITPNM3	-0,60655472097312	NA	NA	NA	DECREASED_H3K27AC_IN_PROMOTER	-2,34348053280945
PKP2	0,340299940008177	NA	NA	NA	INCREASED_H3K27AC_IN_ENHANCER	2,29565746094815
PLA2G7	NA	DECREASED_H3K27AC_IN_ENHANCER	-1,50793274857303	NA	DECREASED_H3K27AC_IN_PROMOTER	-2,71635766093198
PLCH2	-0,42964412089998	NA	NA	NA	DECREASED_H3K27AC_IN_PROMOTER	-2,07578754431605
PLEKHA7	-0,66323259804112	NA	NA	NA	DECREASED_H3K27AC_IN_PROMOTER	-1,96442712233743
PLIN4	-1,00534359385836	NA	NA	NA	DECREASED_H3K27AC_IN_PROMOTER	-2,28126091762964
PLK2	0,372003015457295	NA	NA	NA	INCREASED_H3K27AC_IN_ENHANCER	2,47482961369818
PNPLA6	-0,37231556886269	NA	NA	NA	DECREASED_H3K27AC_IN_PROMOTER	-2,55617267764659
PNRC1	0,35391785599353	NA	NA	0,332552014109102	INCREASED_H3K27AC_IN_ENHANCER	2,13487753191774

PODXL	-0,52015544290092	NA	NA	NA	DECREASED_H3K27AC_IN_PROMOTER	-2,27940072505054
POLDIP3	NA	INCREASED_H3K27AC_IN_PROMOTER	1,58263490235224	NA	INCREASED_H3K27AC_IN_ENHANCER	2,04681515083441
POM121L9P	NA	INCREASED_H3K27AC_IN_ENHANCER	NA	NA	INCREASED_H3K27AC_IN_ENHANCER	2,20816330285762
PPARA	-0,70413194844629	NA	NA	NA	DECREASED_H3K27AC_IN_PROMOTER	-2,58877070580113
PPM1L	-0,45166304931683	NA	NA	NA	DECREASED_H3K27AC_IN_PROMOTER	-1,02698226407985
PPP1R16B	-3,23735084256361	NA	NA	-3,25137866828268	NA	NA
PRDM1	0,546498295309526	NA	NA	NA	INCREASED_H3K27AC_IN_ENHANCER	2,49571622941085
PRR15L	-0,50256634179881	NA	NA	NA	DECREASED_H3K27AC_IN_ENHANCER	-2,30927404817584
PRRC2B	-0,43870370455966	NA	NA	NA	DECREASED_H3K27AC_IN_ENHANCER	-2,54076164997189
PSMC6	0,375705371670813	NA	NA	NA	INCREASED_H3K27AC_IN_PROMOTER_AND_ENHANCER	2,49068702224108
PTGS2	1,46561482587445	NA	NA	NA	INCREASED_H3K27AC_IN_ENHANCER	2,50767916918051
PTP4A1	0,998562228468908	NA	NA	NA	INCREASED_H3K27AC_IN_PROMOTER	2,53415336100025
PTPN23	-0,32213214879755	NA	NA	NA	DECREASED_H3K27AC_IN_PROMOTER	-2,40629173372689
PTPRF	-0,32624394041774	NA	NA	NA	DECREASED_H3K27AC_IN_PROMOTER	-2,3611360546096
PUM1	-0,29703589017582	NA	NA	NA	DECREASED_H3K27AC_IN_ENHANCER	-2,52045503464633
PWWP2B	-0,42120857485506	NA	NA	NA	DECREASED_H3K27AC_IN_ENHANCER	-2,27675351027216
RAB11FIP4	-0,47759097306702	NA	NA	NA	DECREASED_H3K27AC_IN_PROMOTER	-2,29126443763567
RAP1GAP2	-0,9203479564667	NA	NA	NA	DECREASED_H3K27AC_IN_PROMOTER	-0,837697810985114
RAPGEF1	-0,3952593349469	NA	NA	NA	DECREASED_H3K27AC_IN_PROMOTER	-2,4754992182536
RASA3	-0,91775137601372	NA	NA	NA	DECREASED_H3K27AC_IN_PROMOTER	-2,37842863339411
RASSF7	0,340496158540613	NA	NA	0,868618419332591	NA	NA
RGS2	1,96993705483588	NA	NA	NA	INCREASED_H3K27AC_IN_PROMOTER	2,33492097595694
RLF	0,535954231311978	NA	NA	NA	INCREASED_H3K27AC_IN_ENHANCER	2,45672603942589
RNF114	NA	DECREASED_H3K27AC_IN_ENHANCER	-2,00397356115951	NA	DECREASED_H3K27AC_IN_ENHANCER	-1,94243981403001
RNF213	-0,61136622629955	NA	NA	NA	DECREASED_H3K27AC_IN_PROMOTER	-2,00314574949873
RNF220	-0,3911418142167	NA	NA	NA	DECREASED_H3K27AC_IN_PROMOTER	-2,43692942978811
RNF39	0,649280871538957	NA	NA	0,914032591229745	NA	NA
RPH3AL	-1,46356534281638	NA	NA	NA	DECREASED_H3K27AC_IN_PROMOTER	-2,22129699160507
RPL19	0,29340997817664	NA	NA	NA	INCREASED_H3K27AC_IN_PROMOTER	1,1120851881304
RPL27A	0,328928164313173	NA	NA	NA	INCREASED_H3K27AC_IN_ENHANCER	2,22036430313451
RPS26	0,410633745331732	NA	NA	NA	INCREASED_H3K27AC_IN_PROMOTER	2,34730943331324
RPS6KA2	-0,28064992997761	NA	NA	NA	DECREASED_H3K27AC_IN_PROMOTER	-2,55279693884076
RSAD1	-0,40000474160142	NA	NA	NA	DECREASED_H3K27AC_IN_PROMOTER	-2,19668571909853
RSRC2	0,335958289381695	NA	NA	NA	INCREASED_H3K27AC_IN_ENHANCER	2,28111240035087
RXRA	-0,32713831641749	NA	NA	NA	DECREASED_H3K27AC_IN_PROMOTER	-1,92602753134846
SAFB	NA	INCREASED_H3K27AC_IN_PROMOTER	2,06318058812251	NA	INCREASED_H3K27AC_IN_ENHANCER	2,31839663476839
SBF1	-0,40118161564314	NA	NA	NA	DECREASED_H3K27AC_IN_PROMOTER	-2,44465348413841
SBNO2	0,34506374369346	NA	NA	1,31234986976917	NA	NA
SC5D	0,427630114408475	NA	NA	NA	INCREASED_H3K27AC_IN_ENHANCER	2,26139832279074
SCRIB	-0,49209956747999	NA	NA	NA	DECREASED_H3K27AC_IN_PROMOTER	-2,47813289153413
SCYGR4	NA	INCREASED_H3K27AC_IN_PROMOTER	0,996507192702794	NA	INCREASED_H3K27AC_IN_ENHANCER	2,30028338270815
SEC16A	-0,29081340486966	NA	NA	NA	DECREASED_H3K27AC_IN_PROMOTER	-2,16320225995841
SEMA4C	-0,4941318315646	NA	NA	NA	DECREASED_H3K27AC_IN_PROMOTER	-2,43948479144517
SERPINB2	0,484111167497166	NA	NA	1,48599564815759	NA	NA
SERPINB7	0,497583278062184	NA	NA	NA	INCREASED_H3K27AC_IN_PROMOTER	2,35793874550987
SGTA	NA	DECREASED_H3K27AC_IN_PROMOTER	-0,899071287324736	NA	DECREASED_H3K27AC_IN_PROMOTER	-2,57136038328775
SHQ1	0,482386437493516	NA	NA	NA	INCREASED_H3K27AC_IN_ENHANCER	2,22938157605548
SLC12A7	-0,43276374411763	NA	NA	NA	DECREASED_H3K27AC_IN_PROMOTER	-2,51434995190279
SLC22A23	-0,75245287019638	NA	NA	NA	DECREASED_H3K27AC_IN_PROMOTER	-2,53814853128565
SLC23A1	-0,82149044893586	NA	NA	NA	DECREASED_H3K27AC_IN_PROMOTER	-2,25808665057414
SLC26A2	-1,18608194239063	NA	NA	-1,25473079973376	NA	NA
SLC2A14	2,97202067341919	NA	NA	NA	INCREASED_H3K27AC_IN_PROMOTER	2,41046207157683
SLC46A1	-0,68647574178731	NA	NA	NA	DECREASED_H3K27AC_IN_PROMOTER	-1,25259015197202
SLC5A3	0,34300301736097	NA	NA	0,741246720630442	NA	NA
SLC6A8	0,645389375379337	NA	NA	1,23475731866926	NA	NA
SLC7A11	1,19012966948855	NA	NA	NA	INCREASED_H3K27AC_IN_PROMOTER_AND_ENHANCER	1,93795197107456
SNX9	0,289469967245919	NA	NA	NA	INCREASED_H3K27AC_IN_ENHANCER	2,42024080803078
SPON2	-0,49133668577162	NA	NA	NA	DECREASED_H3K27AC_IN_PROMOTER	-2,47794260799847
SQOR	0,356623427509787	NA	NA	NA	INCREASED_H3K27AC_IN_PROMOTER	2,54030838625818

SRSF2	NA	DECREASED_H3K27AC_IN_ENHANCER	-1,7973380590322	NA	DECREASED_H3K27AC_IN_ENHANCER	-2,57070327120275
STC1	2,6288001823272	NA	NA	NA	INCREASED_H3K27AC_IN_ENHANCER	2,39265740427838
SYNE1	-0,79093244452383	NA	NA	NA	DECREASED_H3K27AC_IN_PROMOTER	-2,23802918301871
SYNPO	NA	DECREASED_H3K27AC_IN_ENHANCER	-1,4901811919319	NA	DECREASED_H3K27AC_IN_ENHANCER	-2,56222462798609
TAL1	NA	INCREASED_H3K27AC_IN_PROMOTER	2,1184110263595	3,56663003731915	NA	NA
TCOF1	-0,37774943842196	NA	NA	NA	DECREASED_H3K27AC_IN_ENHANCER	-2,64969997526906
TFCP2L1	-0,58954744588815	NA	NA	-2,45679805543174	NA	NA
TGM2	-1,02243883754546	NA	NA	NA	DECREASED_H3K27AC_IN_PROMOTER_AND_ENHANCER	-2,19010194563547
THEM6	-0,40347024436377	NA	NA	NA	DECREASED_H3K27AC_IN_PROMOTER	-2,5554807733763
THSD4	-0,82058356546948	NA	NA	-0,752240598665205	NA	NA
TJP2	0,344541223066179	NA	NA	NA	INCREASED_H3K27AC_IN_PROMOTER	2,21758090805786
TLE3	-0,36910486037141	NA	NA	NA	DECREASED_H3K27AC_IN_ENHANCER	-2,5056453053004
TLE4	-0,70898691102054	NA	NA	NA	DECREASED_H3K27AC_IN_PROMOTER	-2,24784270333158
TLNRD1	0,368875811856262	NA	NA	1,04656234662665	NA	NA
TMEM156	1,5619517900691	NA	NA	NA	INCREASED_H3K27AC_IN_ENHANCER	2,35978839141763
TMEM187	NA	DECREASED_H3K27AC_IN_ENHANCER	-1,662160651824	NA	DECREASED_H3K27AC_IN_ENHANCER	-2,57883968032348
TMEM201	-0,60059495549692	NA	NA	NA	DECREASED_H3K27AC_IN_PROMOTER	-2,43692942978811
TMEM44	-0,61756684392643	NA	NA	NA	DECREASED_H3K27AC_IN_ENHANCER	-2,62088591842614
TMEM45A	0,600657072212826	NA	NA	1,90245711442474	NA	NA
TMEM63A	-0,46580803035013	NA	NA	NA	DECREASED_H3K27AC_IN_PROMOTER	-2,20019134572342
TNFAIP2	1,14320380807256	NA	NA	NA	INCREASED_H3K27AC_IN_ENHANCER	2,58154033025994
TNFAIP3	0,885369318362706	NA	NA	NA	INCREASED_H3K27AC_IN_PROMOTER	2,32353150800356
TNFAIP8	0,410228016367451	NA	NA	NA	INCREASED_H3K27AC_IN_PROMOTER	2,52325894336454
TNFRSF10A	0,62006779491646	NA	NA	1,33377625439979	NA	NA
TNFRSF10A-DT	0,798392729879901	NA	NA	1,96182037115604	NA	NA
TNPO2	NA	DECREASED_H3K27AC_IN_PROMOTER	-1,18822862203468	NA	DECREASED_H3K27AC_IN_PROMOTER	-2,34078512849791
TNS1	0,85522383706617	INCREASED_H3K27AC_IN_PROMOTER	1,09506810346631	NA	INCREASED_H3K27AC_IN_PROMOTER	2,19040807878454
TNS4	-1,33446435186875	NA	NA	NA	DECREASED_H3K27AC_IN_ENHANCER	-2,35244580616251
TPK1	NA	INCREASED_H3K27AC_IN_ENHANCER	0,859415861373113	NA	INCREASED_H3K27AC_IN_ENHANCER	1,80304410993923
TRA2B	0,481267829571061	NA	NA	NA	INCREASED_H3K27AC_IN_ENHANCER	2,23695814951156
TRAF5	-0,81638468686311	NA	NA	NA	DECREASED_H3K27AC_IN_PROMOTER	-1,87229750609781
TRIM65	-0,46547233425307	DECREASED_H3K27AC_IN_ENHANCER	-1,7973380590322	NA	DECREASED_H3K27AC_IN_PROMOTER_AND_ENHANCER	-2,49366083634204
TRIML2	1,50669267013022	NA	NA	NA	INCREASED_H3K27AC_IN_PROMOTER	2,21225429872627
TRIP4	0,356939687529018	NA	NA	NA	INCREASED_H3K27AC_IN_PROMOTER	2,41526470665458
TTC7A	-0,60418395467434	NA	NA	NA	DECREASED_H3K27AC_IN_PROMOTER	-2,38109467960272
UBALD2	-0,43777519978921	DECREASED_H3K27AC_IN_ENHANCER	-1,7973380590322	NA	DECREASED_H3K27AC_IN_ENHANCER	-2,57070327120275
UBC	0,480082945211415	NA	NA	NA	INCREASED_H3K27AC_IN_PROMOTER_AND_ENHANCER	1,63818408589997
UBE2N	0,465995130867978	NA	NA	NA	INCREASED_H3K27AC_IN_ENHANCER	2,49136568660648
UBR5	-0,34424786197141	NA	NA	NA	DECREASED_H3K27AC_IN_ENHANCER	-2,50705908708156
UGCG	0,3225522238431	NA	NA	NA	INCREASED_H3K27AC_IN_ENHANCER	2,41427141925711
ULBP2	0,452871273668683	NA	NA	1,15119541744736	NA	NA
UNK	NA	DECREASED_H3K27AC_IN_ENHANCER	-1,7973380590322	NA	DECREASED_H3K27AC_IN_ENHANCER	-2,57070327120275
UVSSA	-0,52907296565851	NA	NA	NA	DECREASED_H3K27AC_IN_PROMOTER	-2,54091890491795
VAMP3	0,433399616206111	NA	NA	NA	INCREASED_H3K27AC_IN_ENHANCER	2,25876181701747
VEGFA	0,357039506499844	NA	NA	NA	INCREASED_H3K27AC_IN_ENHANCER	0,805732726069365
VSTM1	NA	INCREASED_H3K27AC_IN_PROMOTER	1,8400104832275	5,20057364343291	NA	NA
WDR27	-0,43493663754509	NA	NA	NA	DECREASED_H3K27AC_IN_PROMOTER	-2,22437509465489
WNT5A	-0,66750040774709	NA	NA	NA	DECREASED_H3K27AC_IN_PROMOTER	-2,54698761899036
WWC1	-0,44545656100983	NA	NA	NA	DECREASED_H3K27AC_IN_PROMOTER	-2,37809202118385
ZBED2	0,77815719111612	NA	NA	NA	INCREASED_H3K27AC_IN_PROMOTER	2,2616340721234
ZBTB16	-1,4619868987911	NA	NA	-3,12407186131286	NA	NA
ZBTB4	-0,36892522747466	NA	NA	NA	DECREASED_H3K27AC_IN_PROMOTER	-2,4114479901791
ZFTA	-0,77703857425386	NA	NA	NA	DECREASED_H3K27AC_IN_PROMOTER	-2,44474420877814
ZNF488	NA	INCREASED_H3K27AC_IN_ENHANCER	1,77215507679238	NA	INCREASED_H3K27AC_IN_ENHANCER	2,50029892125491
ZNF678	NA	INCREASED_H3K27AC_IN_PROMOTER	1,9662702291212	NA	INCREASED_H3K27AC_IN_ENHANCER	2,55164893925002
ZNF827	-0,71453118095406	NA	NA	NA	DECREASED_H3K27AC_IN_PROMOTER	-2,22840634636138
ZNRF1	-0,61731482494481	NA	NA	NA	DECREASED_H3K27AC_IN_PROMOTER	-2,2277597632763
