# Supplementary material for: Airborne fine particulate matter exposure induces transcriptomic alterations resembling asthmatic signatures: insights from integrated omics analysis
Source: Environ Epigenet. 2025 Jan 2;11(1):dvae026. doi: 10.1093/eep/dvae026 (PMC11753294; doi:10.1093/eep/dvae026)
Supplement: dvae026_Supp [file dvae026_supp.zip › suppl_data/Suppltable2.pdf]

Supplementary Table 2. Complete results of ORA analysis of biological processes associated with the 349 common DEGs/DERs.

ID	Description	GeneRatio	BgRatio	pvalue	p.adjust	qvalue	geneID	Count
GO:0001666	response to hypoxia	21/308	298/18870	2,20E-08	7,54E-05	6,02E-05	AK4/ANGPTL4/EDN1/EGLN3/EGR1/EP300/EPHA4/ERO1A/FOS/FOSL2/HIPK2/HK2/HSD11B2/HSPG2/IL1A/PDK1/PGF/PPARA/PTGS2/STC1/VEGFA	21
GO:0070482	response to oxygen levels	22/308	343/18870	5,44E-08	7,54E-05	6,02E-05	AK4/ANGPTL4/EDN1/EGLN3/EGR1/EP300/EPHA4/ERO1A/FOS/FOSL2/GUCY1B1/HIPK2/HK2/HSD11B2/HSPG2/IL1A/PDK1/PGF/PPARA/PTGS2/STC1/VEGFA	22
GO:0036293	response to decreased oxygen levels	21/308	315/18870	5,71E-08	7,54E-05	6,02E-05	AK4/ANGPTL4/EDN1/EGLN3/EGR1/EP300/EPHA4/ERO1A/FOS/FOSL2/HIPK2/HK2/HSD11B2/HSPG2/IL1A/PDK1/PGF/PPARA/PTGS2/STC1/VEGFA	21
GO:0071222	cellular response to lipopolysaccharide	17/308	226/18870	1,96E-07	1,94E-04	1,55E-04	CD274/CSF3/CTR9/CXCL2/CXCL3/CXCL8/DAB2IP/EPHB2/GIT1/IL1A/IL1B/NFKBIZ/PDCD4/SBNO2/SPON2/TNFAIP3/WNT5A	17
GO:0032496	response to lipopolysaccharide	21/308	348/18870	3,04E-07	2,41E-04	1,92E-04	CD274/CSF3/CTR9/CXCL2/CXCL3/CXCL8/DAB2IP/EDN1/EPHB2/FOS/FOSL2/GIT1/IL1A/IL1B/NFKBIZ/PDCD4/PTGS2/SBNO2/SPON2/TNFAIP3/WNT5A	21
GO:0071219	cellular response to molecule of bacterial origin	17/308	239/18870	4,35E-07	2,71E-04	2,16E-04	CD274/CSF3/CTR9/CXCL2/CXCL3/CXCL8/DAB2IP/EPHB2/GIT1/IL1A/IL1B/NFKBIZ/PDCD4/SBNO2/SPON2/TNFAIP3/WNT5A	17
GO:0002064	epithelial cell development	16/308	214/18870	4,90E-07	2,71E-04	2,16E-04	ARID4B/ASXL1/FOSL2/GPR4/GRHL2/IL1A/IL1B/PODXL/PPP1R16B/PRDM1/RAPGEF1/STC1/TFCP2L1/TJP2/VEGFA/WNT5A	16
GO:0048545	response to steroid hormone	20/308	330/18870	5,47E-07	2,71E-04	2,16E-04	CYP1B1/EDN1/EP300/FOS/FOSL2/HSD11B2/IL1RN/KANK2/KMT2D/NCOA4/NCOR2/NR4A3/PER1/PPARA/PTGS2/RXRA/SAFB/STC1/TRIP4/UBR5	20
GO:0002237	response to molecule of bacterial origin	21/308	369/18870	7,93E-07	3,49E-04	2,79E-04	CD274/CSF3/CTR9/CXCL2/CXCL3/CXCL8/DAB2IP/EDN1/EPHB2/FOS/FOSL2/GIT1/IL1A/IL1B/NFKBIZ/PDCD4/PTGS2/SBNO2/SPON2/TNFAIP3/WNT5A	21
GO:0071383	cellular response to steroid hormone stimulus	15/308	208/18870	1,76E-06	6,56E-04	5,24E-04	CYP1B1/EDN1/EP300/KANK2/KMT2D/NCOA4/NCOR2/NR4A3/PER1/PPARA/RXRA/SAFB/STC1/TRIP4/UBR5	15
GO:0071216	cellular response to biotic stimulus	17/308	265/18870	1,82E-06	6,56E-04	5,24E-04	CD274/CSF3/CTR9/CXCL2/CXCL3/CXCL8/DAB2IP/EPHB2/GIT1/IL1A/IL1B/NFKBIZ/PDCD4/SBNO2/SPON2/TNFAIP3/WNT5A	17
GO:0043401	steroid hormone mediated signaling pathway	12/308	138/18870	2,81E-06	9,28E-04	7,41E-04	EP300/KANK2/KMT2D/NCOA4/NCOR2/NR4A3/PER1/PPARA/RXRA/SAFB/TRIP4/UBR5	12
GO:0045765	regulation of angiogenesis	19/308	349/18870	5,10E-06	1,54E-03	1,23E-03	ADAMTS1/ANGPTL4/CXCL8/CYP1B1/DAB2IP/GPR4/HIPK2/HK2/HSPG2/IL1A/IL1B/ITGB3/KLF4/PGF/PLK2/PPP1R16B/TNFAIP3/VEGFA/WNT5A	19
GO:0071456	cellular response to hypoxia	12/308	147/18870	5,43E-06	1,54E-03	1,23E-03	AK4/EDN1/EGLN3/EGR1/EPHA4/ERO1A/FOS/HIPK2/PDK1/PTGS2/STC1/VEGFA	12
GO:1901342	regulation of vasculature development	19/308	354/18870	6,26E-06	1,65E-03	1,32E-03	ADAMTS1/ANGPTL4/CXCL8/CYP1B1/DAB2IP/GPR4/HIPK2/HK2/HSPG2/IL1A/IL1B/ITGB3/KLF4/PGF/PLK2/PPP1R16B/TNFAIP3/VEGFA/WNT5A	19
GO:0198738	cell-cell signaling by wnt	22/308	461/18870	7,67E-06	1,90E-03	1,52E-03	CELSR2/CSNK1D/CTR9/DAB2IP/DVL1/EDN1/EGR1/FAM53B/GRK6/HIC1/JRK/KLF4/LRP1/NFATC4/PTPN23/RNF213/RNF220/TLE3/TLE4/TNFAIP3/UBR5/WNT5A	22
GO:0036294	cellular response to decreased oxygen levels	12/308	158/18870	1,14E-05	2,65E-03	2,12E-03	AK4/EDN1/EGLN3/EGR1/EPHA4/ERO1A/FOS/HIPK2/PDK1/PTGS2/STC1/VEGFA	12
GO:0007369	gastrulation	13/308	197/18870	2,23E-05	4,63E-03	3,69E-03	ATOH8/CTR9/DVL1/IL1RN/ITGB3/ITGB4/KLF4/LAMB3/MEGF8/NR4A3/PHLDB1/TAL1/WNT5A	13
GO:0016055	Wnt signaling pathway	21/308	459/18870	2,32E-05	4,63E-03	3,69E-03	CELSR2/CSNK1D/CTR9/DAB2IP/DVL1/EDN1/EGR1/FAM53B/GRK6/HIC1/JRK/KLF4/LRP1/NFATC4/RNF213/RNF220/TLE3/TLE4/TNFAIP3/UBR5/WNT5A	21
GO:0031334	positive regulation of protein-containing complex assembly	13/308	198/18870	2,36E-05	4,63E-03	3,69E-03	CCL26/CDC42EP5/CDK5RAP2/CRACD/CSF3/DAB2IP/GIT1/NCK2/PSMC6/SNX9/TAL1/TRIM65/VEGFA	13
GO:0001667	ameboidal-type cell migration	22/308	497/18870	2,45E-05	4,63E-03	3,69E-03	ANLN/ATOH8/CYP1B1/DAB2IP/DNAJA4/EDN1/HBEGF/IRS2/ITGB3/ITGB4/KANK2/KLF4/MEGF8/PLK2/PTGS2/PTPN23/SEMA4C/STC1/TMEM201/TNS1/VEGFA/WNT5A	22
GO:0071453	cellular response to oxygen levels	12/308	174/18870	2,99E-05	5,38E-03	4,30E-03	AK4/EDN1/EGLN3/EGR1/EPHA4/ERO1A/FOS/HIPK2/PDK1/PTGS2/STC1/VEGFA	12
GO:0032535	regulation of cellular component size	18/308	370/18870	4,05E-05	6,98E-03	5,57E-03	CAPZB/CCL26/CDC42EP5/CLN8/CRACD/CSF3/DEPTOR/EDN1/IFRD1/KANK2/MEGF8/NCK2/RAP1GAP2/SEMA4C/SLC12A7/SNX9/VEGFA/WNT5A	18
GO:0045732	positive regulation of protein catabolic process	13/308	210/18870	4,35E-05	7,19E-03	5,74E-03	CSNK1D/DAB2IP/DVL1/GGA3/IER3/IL1B/LRP1/PLK2/PSMC6/SGTA/SNX9/TNFAIP3/WNT5A	13
GO:0090132	epithelium migration	18/308	375/18870	4,83E-05	7,31E-03	5,83E-03	ANLN/ATOH8/CYP1B1/DAB2IP/DNAJA4/EDN1/GRHL2/HBEGF/IRS2/ITGB3/KANK2/KLF4/PLK2/PTGS2/PTPN23/STC1/VEGFA/WNT5A	18
GO:0045923	positive regulation of fatty acid metabolic process	6/308	41/18870	5,01E-05	7,31E-03	5,83E-03	CPT1A/IL1B/IRS2/NR4A3/PPARA/PTGS2	6
GO:0007566	embryo implantation	7/308	60/18870	5,31E-05	7,31E-03	5,83E-03	HSPG2/IL1B/ITGB3/ITGB4/PTGS2/STC1/VEGFA	7
GO:0045766	positive regulation of angiogenesis	12/308	185/18870	5,45E-05	7,31E-03	5,83E-03	ANGPTL4/CXCL8/CYP1B1/HIPK2/HK2/IL1A/IL1B/ITGB3/KLF4/PGF/VEGFA/WNT5A	12
GO:1904018	positive regulation of vasculature development	12/308	185/18870	5,45E-05	7,31E-03	5,83E-03	ANGPTL4/CXCL8/CYP1B1/HIPK2/HK2/IL1A/IL1B/ITGB3/KLF4/PGF/VEGFA/WNT5A	12
GO:0032970	regulation of actin filament-based process	18/308	379/18870	5,53E-05	7,31E-03	5,83E-03	ARHGEF10L/ARPIN/ASAP3/CAPZB/CCL26/CDC42EP5/CRACD/CSF3/EDN1/IL1A/ITGB3/KANK2/LRP1/NCK2/PKP2/SNX9/STC1/SYNPO	18
GO:0090130	tissue migration	18/308	380/18870	5,72E-05	7,31E-03	5,84E-03	ANLN/ATOH8/CYP1B1/DAB2IP/DNAJA4/EDN1/GRHL2/HBEGF/IRS2/ITGB3/KANK2/KLF4/PLK2/PTGS2/PTPN23/STC1/VEGFA/WNT5A	18
GO:0043254	regulation of protein-containing complex assembly	19/308	419/18870	6,41E-05	7,68E-03	6,13E-03	ANKRA2/CAMSAP1/CAPZB/CCL26/CDC42EP5/CDK5RAP2/CRACD/CSF3/DAB2IP/EP300/GIT1/KANK2/NCK2/PSMC6/SNX9/TAL1/TRIM65/VEGFA/ZNF827	19
GO:0022407	regulation of cell-cell adhesion	21/308	493/18870	6,52E-05	7,68E-03	6,13E-03	CD274/CELSR2/IL1A/IL1B/IL1RN/IL2RG/ITPKB/KLF4/MAD1L1/MAP3K8/NCK2/NFKBIZ/NR4A3/PAG1/PODXL/PPARA/PTPN23/SCRIB/VEGFA/WNT5A/ZBTB16	21
GO:0045860	positive regulation of protein kinase activity	15/308	282/18870	6,59E-05	7,68E-03	6,13E-03	ADRB2/CSF1/DAB2IP/DLG3/EDN1/EGR1/EPGN/EPHA4/EREG/IL1B/ITGB3/SNX9/TNFRSF10A/VEGFA/WNT5A	15
GO:0030522	intracellular receptor signaling pathway	17/308	353/18870	7,50E-05	8,49E-03	6,78E-03	ASXL1/EP300/GRAMD4/KANK2/KMT2D/MN1/NCOA4/NCOR2/NR4A3/PER1/PPARA/PUM1/RXRA/SAFB/TNFAIP3/TRIP4/UBR5	17
GO:0009755	hormone-mediated signaling pathway	12/308	193/18870	8,19E-05	8,82E-03	7,04E-03	EP300/KANK2/KMT2D/NCOA4/NCOR2/NR4A3/PER1/PPARA/RXRA/SAFB/TRIP4/UBR5	12
GO:0046321	positive regulation of fatty acid oxidation	4/308	15/18870	8,24E-05	8,82E-03	7,04E-03	CPT1A/IRS2/NR4A3/PPARA	4
GO:0032273	positive regulation of protein polymerization	8/308	91/18870	1,21E-04	1,23E-02	9,86E-03	CCL26/CDC42EP5/CDK5RAP2/CRACD/CSF3/GIT1/NCK2/SNX9	8
GO:0010565	regulation of cellular ketone metabolic process	10/308	144/18870	1,30E-04	1,23E-02	9,86E-03	CPT1A/EGR1/IL1B/IRS2/NCOR2/NR4A3/PDK1/PPARA/PTGS2/SLC7A11	10
GO:2000191	regulation of fatty acid transport	5/308	31/18870	1,34E-04	1,23E-02	9,86E-03	EDN1/IL1A/IL1B/IRS2/PPARA	5
GO:0031649	heat generation	4/308	17/18870	1,40E-04	1,23E-02	9,86E-03	ADRB2/IL1A/IL1B/PTGS2	4
GO:0032310	prostaglandin secretion	4/308	17/18870	1,40E-04	1,23E-02	9,86E-03	EDN1/IL1A/IL1B/PTGS2	4
GO:0072109	glomerular mesangium development	4/308	17/18870	1,40E-04	1,23E-02	9,86E-03	EGR1/GPR4/ITGB3/SERPINB7	4
GO:0010631	epithelial cell migration	17/308	372/18870	1,41E-04	1,23E-02	9,86E-03	ANLN/ATOH8/CYP1B1/DAB2IP/DNAJA4/EDN1/HBEGF/IRS2/ITGB3/KANK2/KLF4/PLK2/PTGS2/PTPN23/STC1/VEGFA/WNT5A	17
GO:1902893	regulation of miRNA transcription	7/308	70/18870	1,43E-04	1,23E-02	9,86E-03	ATOH8/EGR1/FOS/KLF4/NCOR2/NFATC4/PPARA	7
GO:0032956	regulation of actin cytoskeleton organization	16/308	337/18870	1,43E-04	1,23E-02	9,86E-03	ARHGEF10L/ARPIN/ASAP3/CAPZB/CCL26/CDC42EP5/CRACD/CSF3/EDN1/IL1A/ITGB3/KANK2/LRP1/NCK2/SNX9/SYNPO	16
GO:1902905	positive regulation of supramolecular fiber organization	11/308	175/18870	1,47E-04	1,24E-02	9,87E-03	ARHGEF10L/CCL26/CDC42EP5/CDK5RAP2/CRACD/CSF3/EDN1/GIT1/NCK2/SNX9/SYNPO	11
GO:0044703	multi-organism reproductive process	12/308	206/18870	1,52E-04	1,25E-02	1,00E-02	EDN1/FOS/HSD11B2/HSPG2/IL1B/ITGB3/ITGB4/PRDM1/PTGS2/RGS2/STC1/VEGFA	12
GO:0061614	miRNA transcription	7/308	71/18870	1,57E-04	1,27E-02	1,01E-02	ATOH8/EGR1/FOS/KLF4/NCOR2/NFATC4/PPARA	7
GO:0051347	positive regulation of transferase activity	18/308	414/18870	1,68E-04	1,33E-02	1,06E-02	ADRB2/CSF1/DAB2IP/DLG3/EDN1/EGR1/EPGN/EPHA4/EREG/GRHL2/IL1B/ITGB3/KLF4/PARN/SNX9/TNFRSF10A/VEGFA/WNT5A	18
GO:0006869	lipid transport	19/308	453/18870	1,77E-04	1,35E-02	1,08E-02	ABCA7/ANO7/ATP10A/CLN8/CPT1A/EDN1/GRAMD1A/IL1A/IL1B/IRS2/ITGB3/LIPG/LRP1/PITPNM2/PITPNM3/PPARA/PTGS2/RXRA/UGCG	19
GO:0030518	intracellular steroid hormone receptor signaling pathway	9/308	122/18870	1,78E-04	1,35E-02	1,08E-02	EP300/KANK2/KMT2D/NCOA4/NCOR2/PER1/SAFB/TRIP4/UBR5	9
GO:0035148	tube formation	10/308	151/18870	1,91E-04	1,43E-02	1,14E-02	ATOH8/DAB2IP/DVL1/GRHL2/PODXL/SCRIB/SEMA4C/TGM2/VEGFA/WNT5A	10
GO:0030838	positive regulation of actin filament polymerization	6/308	52/18870	1,95E-04	1,43E-02	1,14E-02	CCL26/CDC42EP5/CRACD/CSF3/NCK2/SNX9	6
GO:0032368	regulation of lipid transport	10/308	153/18870	2,13E-04	1,53E-02	1,22E-02	ABCA7/EDN1/IL1A/IL1B/IRS2/ITGB3/LIPG/LRP1/PPARA/RXRA	10
GO:1905952	regulation of lipid localization	11/308	183/18870	2,17E-04	1,53E-02	1,22E-02	ABCA7/EDN1/EHD1/IL1A/IL1B/IRS2/ITGB3/LIPG/LRP1/PPARA/RXRA	11
GO:0051495	positive regulation of cytoskeleton organization	11/308	184/18870	2,27E-04	1,58E-02	1,26E-02	ARHGEF10L/CCL26/CDC42EP5/CDK5RAP2/CRACD/CSF3/EDN1/GIT1/NCK2/SNX9/SYNPO	11
GO:0045444	fat cell differentiation	13/308	248/18870	2,31E-04	1,58E-02	1,26E-02	ADGRF1/ADRB2/ASXL1/EP300/ERO1A/FOSL2/KLF4/LAMB3/NR4A3/PTGS2/RGS2/WNT5A/ZBTB16	13
GO:0044706	multi-multicellular organism process	12/308	216/18870	2,36E-04	1,58E-02	1,26E-02	EDN1/FOS/HSD11B2/HSPG2/IL1B/ITGB3/ITGB4/PRDM1/PTGS2/RGS2/STC1/VEGFA	12
GO:0007565	female pregnancy	11/308	186/18870	2,50E-04	1,65E-02	1,32E-02	FOS/HSD11B2/HSPG2/IL1B/ITGB3/ITGB4/PRDM1/PTGS2/RGS2/STC1/VEGFA	11
GO:0006935	chemotaxis	19/308	468/18870	2,67E-04	1,73E-02	1,38E-02	ARHGEF16/CCL26/CSF1/CXCL2/CXCL3/CXCL8/CXCR6/EDN1/HBEGF/IL1B/ITGB3/LSP1/MEGF8/PGF/PLA2G7/SCRIB/SEMA4C/VEGFA/WNT5A	19
GO:0042330	taxis	19/308	470/18870	2,82E-04	1,80E-02	1,44E-02	ARHGEF16/CCL26/CSF1/CXCL2/CXCL3/CXCL8/CXCR6/EDN1/HBEGF/IL1B/ITGB3/LSP1/MEGF8/PGF/PLA2G7/SCRIB/SEMA4C/VEGFA/WNT5A	19
GO:0033280	response to vitamin D	5/308	37/18870	3,18E-04	2,00E-02	1,60E-02	KANK2/MN1/PTGS2/RXRA/STC1	5
GO:0010586	miRNA metabolic process	8/308	105/18870	3,26E-04	2,02E-02	1,61E-02	ATOH8/EGR1/FOS/KLF4/NCOR2/NFATC4/PARN/PPARA	8
GO:0010632	regulation of epithelial cell migration	14/308	295/18870	3,72E-04	2,23E-02	1,78E-02	ATOH8/DAB2IP/DNAJA4/EDN1/HBEGF/IRS2/ITGB3/KLF4/PLK2/PTGS2/PTPN23/STC1/VEGFA/WNT5A	14
GO:0051056	regulation of small GTPase mediated signal transduction	14/308	295/18870	3,72E-04	2,23E-02	1,78E-02	ARHGEF10L/ARHGEF17/CSF1/DAB2IP/DENND3/EPHB2/GIT1/GPR4/ITPKB/KANK2/OBSCN/RAP1GAP2/RASA3/TGM2	14
GO:0048732	gland development	18/308	443/18870	3,82E-04	2,24E-02	1,79E-02	ALDH1A3/ASXL1/CDK5RAP3/CPT1A/CSF1/CYP1B1/EDN1/HIPK2/HK2/IRS2/MAD1L1/NCOR2/SCRIB/TFCP2L1/TGM2/TNFAIP3/VEGFA/WNT5A	18
GO:0045785	positive regulation of cell adhesion	19/308	482/18870	3,85E-04	2,24E-02	1,79E-02	CCDC80/CD274/CSF1/EPHA4/IL1A/IL1B/IL2RG/ITGB3/ITPKB/MAP3K8/NCK2/NFKBIZ/NR4A3/PODXL/PTPN23/TGM2/VEGFA/WNT5A/ZBTB16	19
GO:0051785	positive regulation of nuclear division	6/308	60/18870	4,30E-04	2,47E-02	1,97E-02	EDN1/EPGN/EREG/IL1A/IL1B/WNT5A	6
GO:0033674	positive regulation of kinase activity	15/308	337/18870	4,56E-04	2,47E-02	1,97E-02	ADRB2/CSF1/DAB2IP/DLG3/EDN1/EGR1/EPGN/EPHA4/EREG/IL1B/ITGB3/SNX9/TNFRSF10A/VEGFA/WNT5A	15
GO:0062012	regulation of small molecule metabolic process	15/308	337/18870	4,56E-04	2,47E-02	1,97E-02	CPT1A/EGR1/EP300/GIT1/IER3/IGFBP3/IL1B/IRS2/ME1/NCOR2/NR4A3/PDK1/PPARA/PTGS2/SLC7A11	15
GO:0010742	macrophage derived foam cell differentiation	5/308	40/18870	4,62E-04	2,47E-02	1,97E-02	CSF1/EP300/ITGB3/PPARA/WNT5A	5
GO:0030111	regulation of Wnt signaling pathway	15/308	338/18870	4,70E-04	2,47E-02	1,97E-02	CSNK1D/DAB2IP/EGR1/FAM53B/HIC1/JRK/LRP1/NFATC4/RNF213/RNF220/TLE3/TLE4/TNFAIP3/UBR5/WNT5A	15
GO:0071347	cellular response to interleukin-1	8/308	111/18870	4,74E-04	2,47E-02	1,97E-02	CCL26/CXCL8/DAB2IP/EDN1/EGR1/GBP1/IL1B/IL1RN	8
GO:1905954	positive regulation of lipid localization	8/308	111/18870	4,74E-04	2,47E-02	1,97E-02	ABCA7/EDN1/EHD1/IL1A/IL1B/LIPG/LRP1/RXRA	8

GO:0070562	regulation of vitamin D receptor signaling pathway	3/308	10/18870	4,75E-04	2,47E-02	1,97E-02	KANK2/MN1/RXRA	3
GO:0032370	positive regulation of lipid transport	7/308	86/18870	5,15E-04	2,54E-02	2,03E-02	ABCA7/EDN1/IL1A/IL1B/LIPG/LRP1/RXRA	7
GO:0033273	response to vitamin	7/308	86/18870	5,15E-04	2,54E-02	2,03E-02	IL1A/KANK2/MN1/NFKBIZ/PTGS2/RXRA/STC1	7
GO:2000628	regulation of miRNA metabolic process	7/308	86/18870	5,15E-04	2,54E-02	2,03E-02	ATOH8/EGR1/FOS/KLF4/NCOR2/NFATC4/PPARA	7
GO:0048660	regulation of smooth muscle cell proliferation	10/308	171/18870	5,18E-04	2,54E-02	2,03E-02	ADAMTS1/EDN1/EREG/HBEGF/IGFBP3/ITGB3/NR4A3/PDCD4/PTGS2/TNFAIP3	10
GO:0090077	foam cell differentiation	5/308	41/18870	5,19E-04	2,54E-02	2,03E-02	CSF1/EP300/ITGB3/PPARA/WNT5A	5
GO:0032271	regulation of protein polymerization	11/308	204/18870	5,47E-04	2,62E-02	2,09E-02	CAMSAP1/CAPZB/CCL26/CDC42EP5/CDK5RAP2/CRACD/CSF3/GIT1/KANK2/NCK2/SNX9	11
GO:0043405	regulation of MAP kinase activity	9/308	142/18870	5,49E-04	2,62E-02	2,09E-02	CDK5RAP3/DAB2IP/EDN1/EPGN/IL1B/PDCD4/RGS2/VEGFA/WNT5A	9
GO:1903034	regulation of response to wounding	10/308	173/18870	5,67E-04	2,67E-02	2,13E-02	EDN1/EPHA4/EPHB2/GIT1/HBEGF/KLF4/MYLK/PTPRF/SERPINB2/TNFAIP3	10
GO:0001885	endothelial cell development	6/308	64/18870	6,10E-04	2,76E-02	2,20E-02	IL1B/PPP1R16B/RAPGEF1/STC1/TJP2/VEGFA	6
GO:0048659	smooth muscle cell proliferation	10/308	175/18870	6,20E-04	2,76E-02	2,20E-02	ADAMTS1/EDN1/EREG/HBEGF/IGFBP3/ITGB3/NR4A3/PDCD4/PTGS2/TNFAIP3	10
GO:0050921	positive regulation of chemotaxis	9/308	145/18870	6,38E-04	2,76E-02	2,20E-02	CCL26/CSF1/CXCL8/EDN1/MEGF8/PGF/PLA2G7/VEGFA/WNT5A	9
GO:0001660	fever generation	3/308	11/18870	6,45E-04	2,76E-02	2,20E-02	IL1A/IL1B/PTGS2	3
GO:0001865	NK T cell differentiation	3/308	11/18870	6,45E-04	2,76E-02	2,20E-02	FOSL2/PRDM1/ZBTB16	3
GO:0032000	positive regulation of fatty acid beta-oxidation	3/308	11/18870	6,45E-04	2,76E-02	2,20E-02	CPT1A/IRS2/PPARA	3
GO:0072124	regulation of glomerular mesangial cell proliferation	3/308	11/18870	6,45E-04	2,76E-02	2,20E-02	EGR1/ITGB3/SERPINB7	3
GO:0007162	negative regulation of cell adhesion	14/308	312/18870	6,48E-04	2,76E-02	2,20E-02	CD274/CYP1B1/EPHA4/EPHB2/GBP1/HSPG2/IL1RN/KLF4/MAD1L1/PAG1/PODXL/PPARA/SCRIB/VEGFA	14
GO:0043409	negative regulation of MAPK cascade	10/308	176/18870	6,48E-04	2,76E-02	2,20E-02	ABCA7/CDK5RAP3/DAB2IP/EPHA4/EPHB2/GBP1/IL1B/PDCD4/PER1/RGS2	10
GO:0050727	regulation of inflammatory response	17/308	425/18870	6,58E-04	2,77E-02	2,21E-02	FNDC4/GIT1/GPR4/IER3/IFI35/IL1B/KLF4/NFKBIZ/PDCD4/PLA2G7/PLK2/PPARA/PTGS2/SBNO2/TNFAIP3/TRIM65/WNT5A	17
GO:0051783	regulation of nuclear division	9/308	146/18870	6,71E-04	2,80E-02	2,23E-02	CDK5RAP2/EDN1/EPGN/EREG/IL1A/IL1B/MAD1L1/RPS6KA2/WNT5A	9
GO:1902903	regulation of supramolecular fiber organization	16/308	389/18870	7,01E-04	2,85E-02	2,28E-02	ARHGEF10L/ARPIN/ASAP3/CAMSAP1/CAPZB/CCL26/CDC42EP5/CDK5RAP2/CRACD/CSF3/EDN1/GIT1/KANK2/NCK2/SNX9/SYNPO	16
GO:0001894	tissue homeostasis	13/308	279/18870	7,09E-04	2,85E-02	2,28E-02	ADRB2/CLN8/CRACD/CSF1/FOSL2/INPP5D/ITGB3/MUC4/NFKBIZ/PTGS2/TJP2/TNFAIP3/VEGFA	13
GO:0060249	anatomical structure homeostasis	13/308	279/18870	7,09E-04	2,85E-02	2,28E-02	ADRB2/CLN8/CRACD/CSF1/FOSL2/INPP5D/ITGB3/MUC4/NFKBIZ/PTGS2/TJP2/TNFAIP3/VEGFA	13
GO:0006090	pyruvate metabolic process	8/308	118/18870	7,12E-04	2,85E-02	2,28E-02	EP300/GIT1/HK2/IER3/ME1/NR4A3/PDK1/PPARA	8
GO:0045840	positive regulation of mitotic nuclear division	5/308	44/18870	7,23E-04	2,86E-02	2,29E-02	EDN1/EPGN/EREG/IL1A/IL1B	5
GO:0140747	regulation of ncRNA transcription	7/308	92/18870	7,72E-04	3,01E-02	2,41E-02	ATOH8/EGR1/FOS/KLF4/NCOR2/NFATC4/PPARA	7
GO:0002687	positive regulation of leukocyte migration	9/308	149/18870	7,76E-04	3,01E-02	2,41E-02	CSF1/CXCL8/EDN1/IL1A/ITGB3/PGF/PLA2G7/VEGFA/WNT5A	9
GO:0070561	vitamin D receptor signaling pathway	3/308	12/18870	8,49E-04	3,23E-02	2,58E-02	KANK2/MN1/RXRA	3
GO:0072110	glomerular mesangial cell proliferation	3/308	12/18870	8,49E-04	3,23E-02	2,58E-02	EGR1/ITGB3/SERPINB7	3
GO:1903829	positive regulation of protein localization	18/308	476/18870	8,80E-04	3,32E-02	2,65E-02	ABCA7/ARHGEF16/CDK5RAP3/DVL1/EP300/EPHB2/FRMD4A/IL1A/IL1B/IRS2/LRP1/MYO18A/PTGS2/PTPN23/RPH3AL/SLC5A3/UBR5/WNT5A	18
GO:0051258	protein polymerization	13/308	286/18870	8,91E-04	3,33E-02	2,66E-02	CAMSAP1/CAPZB/CCDC57/CCL26/CDC42EP5/CDK5RAP2/CRACD/CSF3/CSNK1D/GIT1/KANK2/NCK2/SNX9	13
GO:0050996	positive regulation of lipid catabolic process	4/308	27/18870	9,08E-04	3,34E-02	2,67E-02	CPT1A/IL1B/IRS2/PPARA	4
GO:0032835	glomerulus development	6/308	69/18870	9,11E-04	3,34E-02	2,67E-02	ASXL1/EGR1/GPR4/ITGB3/PODXL/SERPINB7	6
GO:0015749	monosaccharide transmembrane transport	8/308	123/18870	9,35E-04	3,40E-02	2,71E-02	EDN1/HK2/IL1B/IRS2/NR4A3/SLC23A1/SLC2A14/SLC5A3	8
GO:0050679	positive regulation of epithelial cell proliferation	11/308	218/18870	9,47E-04	3,41E-02	2,72E-02	CCL26/EPGN/HSPG2/IRS2/ITGB3/NR4A3/PGF/PPP1R16B/TNFAIP3/VEGFA/WNT5A	11
GO:0060326	cell chemotaxis	14/308	325/18870	9,62E-04	3,43E-02	2,74E-02	ARHGEF16/CCL26/CSF1/CXCL2/CXCL3/CXCL8/CXCR6/EDN1/HBEGF/IL1B/PGF/PLA2G7/VEGFA/WNT5A	14
GO:0002690	positive regulation of leukocyte chemotaxis	7/308	96/18870	9,94E-04	3,52E-02	2,81E-02	CSF1/CXCL8/EDN1/PGF/PLA2G7/VEGFA/WNT5A	7
GO:0007584	response to nutrient	9/308	155/18870	1,03E-03	3,54E-02	2,83E-02	CYP1B1/IL1A/KANK2/LIPG/MN1/NFKBIZ/PTGS2/RXRA/STC1	9
GO:0008361	regulation of cell size	10/308	187/18870	1,03E-03	3,54E-02	2,83E-02	CLN8/DEPTOR/EDN1/IFRD1/MEGF8/RAP1GAP2/SEMA4C/SLC12A7/VEGFA/WNT5A	10
GO:0015732	prostaglandin transport	4/308	28/18870	1,05E-03	3,54E-02	2,83E-02	EDN1/IL1A/IL1B/PTGS2	4
GO:0032104	regulation of response to extracellular stimulus	4/308	28/18870	1,05E-03	3,54E-02	2,83E-02	KANK2/MN1/PPARA/RXRA	4
GO:0032107	regulation of response to nutrient levels	4/308	28/18870	1,05E-03	3,54E-02	2,83E-02	KANK2/MN1/PPARA/RXRA	4
GO:0032306	regulation of prostaglandin secretion	3/308	13/18870	1,09E-03	3,60E-02	2,87E-02	EDN1/IL1A/IL1B	3
GO:0032308	positive regulation of prostaglandin secretion	3/308	13/18870	1,09E-03	3,60E-02	2,87E-02	EDN1/IL1A/IL1B	3
GO:0045986	negative regulation of smooth muscle contraction	3/308	13/18870	1,09E-03	3,60E-02	2,87E-02	ADRB2/PTGS2/RGS2	3
GO:0048661	positive regulation of smooth muscle cell proliferation	7/308	98/18870	1,12E-03	3,68E-02	2,93E-02	ADAMTS1/EDN1/EREG/HBEGF/ITGB3/NR4A3/PTGS2	7
GO:0051384	response to glucocorticoid	8/308	127/18870	1,15E-03	3,74E-02	2,98E-02	CYP1B1/EDN1/FOS/FOSL2/HSD11B2/IL1RN/PTGS2/STC1	8
GO:0010976	positive regulation of neuron projection development	9/308	158/18870	1,18E-03	3,79E-02	3,02E-02	DAB2IP/DVL1/EHD1/EP300/EPHB2/RAPGEF1/RGS2/VEGFA/WNT5A	9
GO:0061028	establishment of endothelial barrier	5/308	49/18870	1,19E-03	3,79E-02	3,03E-02	IL1B/PPP1R16B/RAPGEF1/TJP2/VEGFA	5
GO:0072012	glomerulus vasculature development	4/308	29/18870	1,20E-03	3,80E-02	3,03E-02	EGR1/GPR4/ITGB3/SERPINB7	4
GO:0001704	formation of primary germ layer	8/308	128/18870	1,21E-03	3,81E-02	3,04E-02	ATOH8/CTR9/ITGB3/ITGB4/KLF4/LAMB3/TAL1/WNT5A	8
GO:0019217	regulation of fatty acid metabolic process	7/308	100/18870	1,26E-03	3,92E-02	3,13E-02	CPT1A/IL1B/IRS2/NR4A3/PDK1/PPARA/PTGS2	7
GO:0042180	cellular ketone metabolic process	11/308	226/18870	1,27E-03	3,92E-02	3,13E-02	CPT1A/EGR1/HSD11B2/IL1B/IRS2/NCOR2/NR4A3/PDK1/PPARA/PTGS2/SLC7A11	11
GO:0010975	regulation of neuron projection development	17/308	453/18870	1,32E-03	4,05E-02	3,24E-02	DAB2IP/DVL1/EHD1/EP300/EPHA4/EPHB2/IFRD1/KLF4/MEGF8/NFATC4/PTPRF/RAPGEF1/RGS2/SEMA4C/SYNE1/VEGFA/WNT5A	17
GO:0010575	positive regulation of vascular endothelial growth factor productio	4/308	30/18870	1,36E-03	4,12E-02	3,29E-02	CYP1B1/IL1A/IL1B/PTGS2	4
GO:0050930	induction of positive chemotaxis	3/308	14/18870	1,37E-03	4,12E-02	3,29E-02	CXCL8/PGF/VEGFA	3
GO:0098760	response to interleukin-7	3/308	14/18870	1,37E-03	4,12E-02	3,29E-02	FOSL2/IL2RG/LSP1	3
GO:0043407	negative regulation of MAP kinase activity	5/308	51/18870	1,43E-03	4,24E-02	3,39E-02	CDK5RAP3/DAB2IP/IL1B/PDCD4/RGS2	5
GO:0010594	regulation of endothelial cell migration	11/308	231/18870	1,51E-03	4,41E-02	3,52E-02	ATOH8/DAB2IP/DNAJA4/EDN1/ITGB3/KLF4/PLK2/PTGS2/STC1/VEGFA/WNT5A	11
GO:0002696	positive regulation of leukocyte activation	15/308	380/18870	1,53E-03	4,41E-02	3,52E-02	CD274/EPHB2/IL1A/IL1B/IL2RG/INPP5D/IRS2/ITPKB/KMT5C/MAP3K8/NCK2/NFKBIZ/NR4A3/WNT5A/ZBTB16	15
GO:0034143	regulation of toll-like receptor 4 signaling pathway	4/308	31/18870	1,55E-03	4,41E-02	3,52E-02	DAB2IP/IFI35/TNFAIP3/ZNRF1	4
GO:0061437	renal system vasculature development	4/308	31/18870	1,55E-03	4,41E-02	3,52E-02	EGR1/GPR4/ITGB3/SERPINB7	4
GO:0061440	kidney vasculature development	4/308	31/18870	1,55E-03	4,41E-02	3,52E-02	EGR1/GPR4/ITGB3/SERPINB7	4
GO:2000273	positive regulation of signaling receptor activity	4/308	31/18870	1,55E-03	4,41E-02	3,52E-02	ADRB2/EDN1/EPGN/EPHB2	4
GO:1903037	regulation of leukocyte cell-cell adhesion	15/308	382/18870	1,62E-03	4,54E-02	3,63E-02	CD274/IL1A/IL1B/IL2RG/ITPKB/KLF4/MAD1L1/MAP3K8/NCK2/NFKBIZ/NR4A3/PAG1/PPARA/SCRIB/ZBTB16	15
GO:0032890	regulation of organic acid transport	6/308	77/18870	1,62E-03	4,54E-02	3,63E-02	EDN1/IL1A/IL1B/IRS2/PPARA/RGS2	6
GO:0044849	estrous cycle	3/308	15/18870	1,69E-03	4,66E-02	3,72E-02	CYP1B1/EGR1/NCOR2	3
GO:1901722	regulation of cell proliferation involved in kidney development	3/308	15/18870	1,69E-03	4,66E-02	3,72E-02	EGR1/ITGB3/SERPINB7	3
GO:0071825	protein-lipid complex organization	5/308	53/18870	1,70E-03	4,66E-02	3,72E-02	ABCA7/LIPG/PCSK6/PLA2G7/SNX9	5
GO:0060055	angiogenesis involved in wound healing	4/308	32/18870	1,75E-03	4,77E-02	3,81E-02	GPR4/ITGB3/TNFAIP3/VEGFA	4
GO:0030833	regulation of actin filament polymerization	8/308	136/18870	1,78E-03	4,77E-02	3,81E-02	CAPZB/CCL26/CDC42EP5/CRACD/CSF3/KANK2/NCK2/SNX9	8
GO:0034219	carbohydrate transmembrane transport	8/308	136/18870	1,78E-03	4,77E-02	3,81E-02	EDN1/HK2/IL1B/IRS2/NR4A3/SLC23A1/SLC2A14/SLC5A3	8
GO:0098781	ncRNA transcription	8/308	136/18870	1,78E-03	4,77E-02	3,81E-02	ATOH8/EGR1/FOS/KLF4/NCOR2/NFATC4/PPARA/TCOF1	8
GO:0110053	regulation of actin filament organization	12/308	273/18870	1,84E-03	4,84E-02	3,86E-02	ARHGEF10L/ARPIN/ASAP3/CAPZB/CCL26/CDC42EP5/CRACD/CSF3/KANK2/NCK2/SNX9/SYNPO	12
GO:0030520	intracellular estrogen receptor signaling pathway	5/308	54/18870	1,84E-03	4,84E-02	3,86E-02	KANK2/KMT2D/NCOA4/SAFB/TRIP4	5
GO:0050873	brown fat cell differentiation	5/308	54/18870	1,84E-03	4,84E-02	3,86E-02	ADRB2/ERO1A/LAMB3/PTGS2/RGS2	5
GO:0007611	learning or memory	12/308	274/18870	1,90E-03	4,95E-02	3,95E-02	AAAS/ABCA7/ADGRF1/CLN8/EP300/EPHB2/FOS/GIT1/NFATC4/PLK2/PTGS2/SLC7A11	12
