# Supplementary material for: Airborne fine particulate matter exposure induces transcriptomic alterations resembling asthmatic signatures: insights from integrated omics analysis
Source: Environ Epigenet. 2025 Jan 2;11(1):dvae026. doi: 10.1093/eep/dvae026 (PMC11753294; doi:10.1093/eep/dvae026)
Supplement: dvae026_Supp [file dvae026_supp.zip › suppl_data/suppltable3.pdf]

Supplementary Table 3. Complete list of hub genes of modules from PM2.5 exposure co-expression network significantly correlated with the exposure and preserved into the asthma diagnosis network.

**Gene**	**Module**	**Intramodular**
CCT6A	blue	93,8902656
MRPL18	blue	92,4302917
CACYBP	blue	91,2529372
DNAJA1	blue	87,2044737
TCP1	blue	85,3695338
DPH3	blue	85,2334583
CNOT10	blue	84,9370404
CCT2	blue	84,4289726
EIF2S1	blue	84,2004137
BUD31	blue	84,0015171
IFT52	blue	81,003804
HSPE1	blue	80,2404138
NRAV	blue	79,9139165
HSPA1L	blue	79,2864689
DDX1	blue	78,6877933
GPN1	blue	77,9167014
ORC5	blue	77,3160158
CHORDC1	blue	76,4517941
HSPD1	blue	75,8280553
UBL5	blue	75,8204208
AHSA1	blue	74,9996663
RBM45	blue	74,9512093
ZNF268	blue	74,6253766
NOP58	blue	74,0470345
MORF4L2	blue	73,8778938
SLC25A46	blue	73,6996576
DNAJC8	blue	73,4705692
TMA7	blue	73,377754
C2orf76	blue	73,1871826
TIMM23	blue	73,1170489
AIMP1	blue	72,846114
FAM13A	blue	72,4526229
LAMTOR3	blue	72,1284976
NUDC	blue	71,9718255
PSMC1	blue	71,8359467
DYNLL1	blue	71,5365916
PSMC6	blue	71,3474671
SUGT1	blue	71,0601523
SNX3	blue	70,8844368
METAP2	blue	70,8474111
SRSF3	blue	70,6926143
COX7A2	blue	70,6257332
MED31	blue	70,1117938
FAIM	blue	69,5260756
DCTN6	blue	69,5048179
CYB5B	blue	69,3270878
GON7	blue	68,3496212
MRPL57	blue	68,2389125
SAP18	blue	67,4881519
IFT22	blue	67,4183568
EIF3J	blue	67,3641399
RDH11	darkolivegreen	11,9001632
PFKFB3	darkolivegreen	11,0526267
YWHAG	darkolivegreen	10,6597271
PLOD2	darkolivegreen	10,4944839
SERPINH1	darkolivegreen	9,71173053
FAM110C	darkolivegreen	9,69645908
IER5	darkolivegreen	9,53817617
TOR1A	darkolivegreen	9,51181106
NOP14,AS1	darkolivegreen	9,14643659
ACAA1	darkolivegreen	8,67375825
ARPC4	darkolivegreen	8,39537288
TGFB2	lightgreen	10,9101642
HIF1A	lightgreen	10,815836
MET	lightgreen	9,8329327
EFNA5	lightgreen	9,81860108
GCNT3	lightgreen	9,70708503
PTPRK	lightgreen	9,40734205
TFPI	lightgreen	9,16094563
JAG1	lightgreen	8,59415484
PTGES	lightgreen	8,56904664
IRAK3	lightgreen	8,42889634
STEAP4	lightgreen	8,30975297
ZMAT3	lightgreen	8,2308142
DST	lightgreen	7,97335279
RHOBTB3	lightgreen	7,81946032
SLC6A14	lightgreen	7,73797343
